# Transformation of 3D Metal–Organic Frameworks into Nanosheets with Enhanced Memristive Behavior for Electronic Data Processing

**DOI:** 10.1002/advs.202405989

**Published:** 2025-03-02

**Authors:** Yuri A. Mezenov, Semyon V. Bachinin, Yuliya A. Kenzhebayeva, Anastasiia S. Efimova, Pavel V. Alekseevskiy, Daria Poloneeva, Anastasia Lubimova, Svyatoslav A. Povarov, Vladimir Shirobokov, Mikhail S. Dunaevskiy, Aleksandra S. Falchevskaya, Andrei S. Potapov, Alexander Novikov, Artem A. Selyutin, Pascal Boulet, Alena N. Kulakova, Valentin A. Milichko

**Affiliations:** ^1^ Qingdao Innovation and Development Center Harbin Engineering University Qingdao Shandong 266000 China; ^2^ School of Physics and Engineering ITMO University St. Petersburg 197101 Russia; ^3^ Advanced Catalytic Materials (ACM) KAUST Catalysis Center (KCC) Division of Physical Sciences and Engineering King Abdullah University of Science and Technology Thuwal 23955 Saudi Arabia; ^4^ ITMO University “Solution Chemistry of Advanced Materials and Technologies” (SCAMT) International Institute Saint Petersburg 191002 Russia; ^5^ Nikolaev Institute of Inorganic Chemistry Siberian Branch of the Russian Academy of Sciences Laboratory of Metal‐Organic Coordination Polymers Novosibirsk 630090 Russia; ^6^ Saint Petersburg State University Saint Petersburg 199034 Russia; ^7^ Рeoples’ Friendship University of Russia Moscow 117198 Russia; ^8^ Institut Jean Lamour Universit de Lorraine UMR CNRS 7198 Nancy 54011 France

**Keywords:** memristive behavior, metal–organic frameworks, nanosheets, structural transformation

## Abstract

The transition from three‐dimensional (3D) to two‐dimensional (2D) semiconducting and insulating materials for micro‐ and opto‐electronics is driven by an energy efficiency and device miniaturization. Herein, the simplicity of growth and stacking of 2D metal–organic framework (MOF) with such planar devices opens up new perspectives in controlling their efficiency and operating parameters. Here, the study reports on 3D to 2D MOF’ structural transformation to achieve ultrathin nanosheets with enhanced insulating properties. Based on neutral N‐donor ligands, the study designs and solvothermally synthesizes 3D MOFs followed by their thermal and solvent treatment to implement the transformation. A set of single crystal and powder X‐ray diffraction, electron microscopy, Raman spectroscopy, numerical modeling, and mechanical exfoliation confirm the nature of the transformation. Compared with initial 3D MOF, its nanosheets demonstrate sufficient changes in electronic properties, expressed as tuning their absorption, photoluminescence, and resistivity. The latter allows to demonstrate the prototype of ultrathin memristive element based on a 4 to 32 nm MOF nanosheet with enhanced functionality (150 to 1400 ON/OFF ratio, retention time exceeding 7300 s, and 100 cycles of switching), thereby, extending the list of scalable and insulating 2D MOFs for micro‐ and opto‐electronics.

## Graphical abstract

The design of 3D MOFs based on neutral and anionic ligands allows implementing the structural transformation from 3D to 2D nanosheets with improved insulating properties, utilized for efficient ultrathin memristive elements.

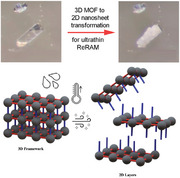

## 1 Introduction

Metal‐organic frameworks (MOFs), being a part of a family of coordination polymers, represent a specific class of materials, consisting of metal nodes or clusters linked to organic ligands in an ordered fashion.^[^ ^1^ ^]^ Organic‐inorganic nature, chemical diversity, and high porosity of MOFs allow one to utilize them for gas storage, separation, sensing, catalysis, and even energy applications.^[^ ^1^, ^2^ ^]^ Intriguing is that the combination of a number of chemical (weak to strong) interactions with organic‐inorganic nature distinguishes MOFs from most inorganic and organic crystals. In contrast to the latter, this combination opens up great possibilities for multi‐stage, diverse, and energy‐efficient structural transformations of MOFs, ranging from classical phase transitions to changing the ligand conformation, isomerization, and rearrangements.^[^ ^3^ ^]^ These transformations became recently as a new tool to control and improve functional properties of MOFs. For example, phase transition from *C_c_* to *C_2_* space group of Zn‐based MOF led to giant enhancement of its nonlinear optical properties.^[^ ^4a^ ^]^ Specific Cu‐based MOFs demonstrated an improved catalytic activity after phase transition (with a change in their crystal symmetry).^[^ ^4b,c^ ^]^ In addition, recent studies also demonstrated that the phase transitions improved the sorption properties of some MOFs.^[^ ^4d,e^ ^]^

However, a number of chemical (weak to strong) interactions allows for more intriguing structural transformations of MOFs (and also covalent‐organic frameworks, COFs), such as changing their dimensionality (D): 3D→2D, 2D→3D, 3D↔2D, 1D→2D→3D, and also 3D→1D, which could be initiated by guest molecules, post synthetic pillar‐ligand insertion, thermal treatment, ligand exchange, and cycloaddition reaction, as well as the variation of the ratio of precursors, and modulators during the MOF synthesis (Table S1, Supporting Information).^[^ ^5^ ^]^ The exfoliation process (including mechanical and sonication) has been also applied for 3D→2D MOF transformation (Table S1, Supporting Information).^[^ ^6^ ^]^ As a result, the changes in the crystal dimensionality significantly affected on the electronic properties of MOFs, and therefore improved (or even formed new) functionality, such as ionic, proton, and electronic conductivities, gas adsorption/separation, surface area, and also light emission.^[^ ^5^, ^7^ ^]^ Moreover, exfoliation of 3D MOFs into 2D nanosheets has been recently demonstrated to enhance catalytic, separation, storage, magnetic, luminescent, and mechanical properties of a new 2D form.^[^ ^6^, ^7^ ^]^

In general, the transition from 3D to 2D insulating or semiconducting materials for micro‐ and optoelectronic technologies is driven by an energy efficiency and miniaturization of the corresponding devices.^[^ ^8^ ^]^ In this sense, 2D MOFs and MOF nanosheets turn out to be competitors to existing inorganic counterparts.^[^ ^2^, ^9^ ^]^ However, the possibility of obtaining the desired structure and dimensionality of MOF, using conventional chemical synthesis, is limited by the features and mechanisms of coordination, nucleation, and growth of MOFs.^[^ ^10^ ^]^ Thus, the post‐synthetic structural transformation of MOFs like 3D→2D can be considered as a new tool to achieve the desired dimensionality (i.e., 2D MOFs and MOF nanosheets).^[^ ^5a,e,i–k^ ^]^

Here we propose 3D→2D MOFs structural transformation through the utilization of neutral N‐donor ligands.^[^ ^11^ ^]^ Initially, we designed and solvothermally synthesized 3D MOFs Zn‐NDC/BPE and Co‐NDC/BPE based on 1,2‐bis(4‐pyridyl)ethylene (BPE) and 2,6‐naphthalenedicarboxylate (NDC) ligands, followed by their thermal and solvent treatment to implement 3D→2D transformation. A set of single crystal and powder X‐ray diffraction, electron microscopy, Raman spectroscopy, numerical modeling, and mechanical exfoliation confirmed the nature of the transformation. Compared with the initial 3D MOF, its nanosheets demonstrated sufficient changes in electronic properties, expressed as tuning their optical absorption (by 0.2 eV), photoluminescence (PL, by 100 nm), and resistivity by 50 times. The latter allowed us to demonstrate the prototype of an ultrathin memristive element based on a 4 to 32 nm MOF nanosheet with enhanced functionality (150 to 1400 ON/OFF ratio, retention time exceeding 7300 s, and 100 cycles of switching), thereby, extending the list of scalable and insulating 2D MOFs for micro‐ and opto‐electronics.

## 2 Results and Discussion

**Figure 1:**
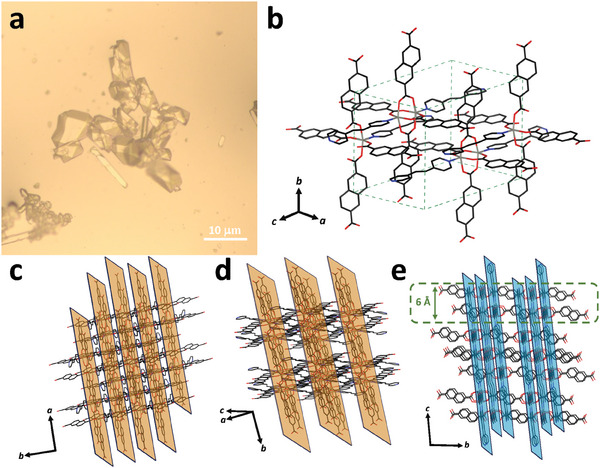
a) The optical image of the as‐synthesized (3D) Zn‐NDC‐BPE MOF (**1**). b) The unit cell of Zn‐NDC/BPE (**1**) (CCDC 2347223). c,d) The visual representation of NDC ligand links along *a* and *b* axes in **1**, respectively. e) The visual representation of BPE ligand links along *c* axis.

The rational design of MOF with a potential 3D→2D structural transformation has been based on the combination of two (neutral and anionic) ligands. For this, 1,2‐bis(4‐pyridyl)ethylene (BPE) and 2,6‐naphthalenedicarboxylic acid (NDC) ligands, and Zn (Co) ions have been chosen to form initial 3D MOFs via conventional solvothermal synthesis (for more details, see Experimental section). Recently, these two ligands has been used to synthesize an interpenetrated 3D Zn‐based MOF with a monoclinic space group *P2_1_/c*, demonstrating a reversible structural transformation via solvent exchange.^[^ ^12^ ^]^ However, in our case, the single crystal X‐ray diffraction analysis (SCXRD, Tables S2 and S3, Supporting Information) revealed that the as‐synthesized MOFs crystalized into triclinic system with a *P‐1* space group (for Zn‐NDC/BPE [Zn_7_(NDC)_2_(BPE)_2_] (1) and for Co‐NDC/BPE [Co_7_(NDC)_2_(BPE)_2_]), possessing the colorless (**Figure** **1**a, for **1**) and pale pink shades (Figure S4, Supporting Information, for Co‐NDC/BPE). Figure 1b also demonstrates that the as‐synthesized MOFs possess the structure, where the bidentate NDC ligand links with the paddle‐wheel metal cluster along *a* and *b* axis, forming 2D layers of 6 Å thickness (Figure 1c,d); while the monodentate neutral BPE ligand serves as a bridge along *c* axis (Figure 1e), connecting these layers into the resulting 3D structure.

Since we have assumed that the monodentate neutral BPE ligand should impair coordination due to less favorable Zn‐N (or Co‐N) bonds in terms of energy (in contrast to Zn‐O bonds with bidentate NDC ligand), we have exposed the as‐synthesized MOFs to external stimuli (drying and washing in solvents). As a result, washing in dimethylformamide (DMF) and keeping of **1** for several days at room temperature led to the crumbling and color changing (from colorless to white, Video S1, Supporting Information). The same behavior has been observed during washing of **1** in DMF followed by fast drying at 30 °C in air. Increasing the temperature up to 120 °C led to the same crumbling effect (see Figure S6, Supporting Information). Observing similar reaction of Zn‐NDC/BPE (**1**) and its isostructural Co‐NDC/BPE on the external stimuli (temperature and solvent), we have selected **1** for further analysis.

**Figure 2:**
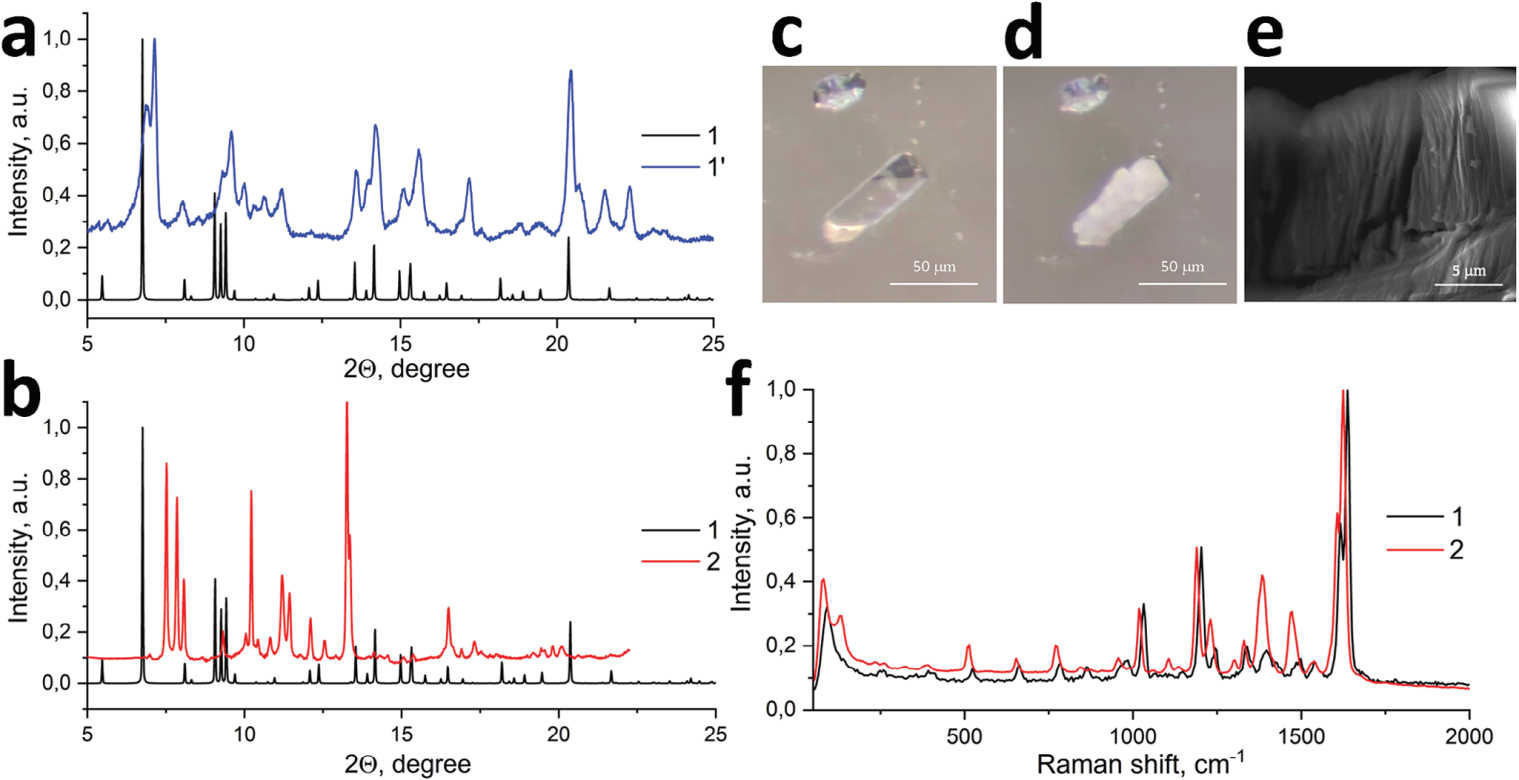
a) PXRD pattern of **1′**, compared with a modeled PXRD pattern of the as‐synthesized MOF **1**. b) PXRD pattern of **2** and modelled PXRD pattern of **1**. c,d) The optical images of the structural transformation from **1** (c) to **2 (**d) over time at 30 °C in air (see also Video S1, Supporting Information). e) SEM micrograph of **2**, confirming the layered morphology. f) Raman spectra of single crystals of **1** and **2**.

**Figure** **2**a,b demonstrates the comparative analysis of the powder X‐ray diffraction (PXRD) for the as‐synthesized 3D MOF (**1**, modeled from the SCXRD data), the powder of **1** washed twice in DMF and quickly dried under vacuum (**1′**), and the powder of **1** washed three times in DMF and then dried in oven at 60 °C for 24 h in air (**2**). As one can see, the PXRD pattern of **1′** matched well with the modeled pattern of **1** (Figure 2a), while **2** demonstrated sufficient mismatch with the modeled PXRD pattern of **1** (Figure 2b). This means, that the as‐synthesized **1** undergoes the structural transformation to **2** upon the action of external stimuli. Herein, the modest drying conditions could prevent this transformation (**1′**, Figure 2a). These results have been also confirmed visually: We have placed the as‐synthesized MOF crystals **1** from the solution (DMF) on a substrate followed by the solvent evaporation in air at 30 °C. Figure 2c,d and Video S1 (Supporting Information) clearly demonstrate the flaking and the crumbling of **1** throughout its volume over time. Additionally, scanning electron microscopy (SEM) confirmed the loss of a monolithic structure of **1**, followed by the flaking (Figure 2e; Figure S8, Supporting Information). Important is that it was quite complicated to perform SCXRD analysis for **2** due to the flaking process. Therefore, we have performed a set of indirect analyzes (PXRD, Raman spectroscopy, mechanical exfoliation, and electron microscopy) to confirm the process of 3D to 2D transformation.^[^ ^6b^ ^]^

First, the analysis of PXRD patterns for **1** and **2** shows not only their fundamental difference (Figure 2a,b), but also the structural difference between **2** and other existing 2D and 3D frameworks based on zinc and NDC ligand (Table S4, Figure S2, Supporting Information). This proves the formation of a new 2D structure after the transformation. Moreover, analysis of the surface area revealed a three‐fold increase of the surface area of **2** (compared with **1**, Table S5, Supporting Information), probably due to the formation of interlayer space (Scheme S1, Supporting Information). X‐Ray photoelectron spectroscopy (XPS) also confirmed some changes in the peak positions and their widths (Figures S9 and S10, Supporting Information), indicating the possible coordination of H_2_O molecules from the air with Zn (instead of the residual BPE ligand).

Second, the confocal Raman spectroscopy for single crystals of **1** and **2** revealed the following (Figure 2f; Figure S16 and Table S8, Supporting Information): (i) The peaks in the range of 520–1032 cm^−1^, attributed mostly to the vibrations of NDC ligand, demonstrated a 20 cm^−1^ shift to lower energies and some narrowing (Table S8).^[^ ^13a,b^ ^]^ (ii) The peaks in the range of 1032–1637 cm^−1^ (corresponding mostly to the vibrations of BPE ligand) demonstrated similar 20 cm^−1^ shift to lower energies, appearance of additional peaks (i.e., 1135 and 1330 cm^−1^), as well as partial broadening and the growth in their intensity (for 1375 and 1460 cm^−1^).^[^ ^13c^ ^]^ (iii) Weakly pronounced peaks in the range of 390–412 cm^−1^ could be assigned to Zn‐N bonds, but it was difficult to track their evolution taking into account the error.^[^ ^13d^ ^]^ Herein, Raman peak at 260 cm^−1^ is associated with Zn‐O bonds, and its intensity can indicate the concentration of nitrogen near Zinc (see Supplementary Information). Indeed, significant decrease of the intensity of this peak for **2**, compared with **1**, could be assigned with impairing the coordination of Zn‐N bond between Zn cluster and BPE ligand, leading to decrease the concentration of nitrogen in the coordination sphere of 2D Zn‐NDC/BPE (**2**). (iv) And finally, the transformation from **1** to **2** has been associated with an appearance of a new peak at 130 cm^−1^ attributed to Zn‐O bond and potential adsorption of H_2_O from the air.^[^ ^13e,f^ ^]^

**Figure 3:**
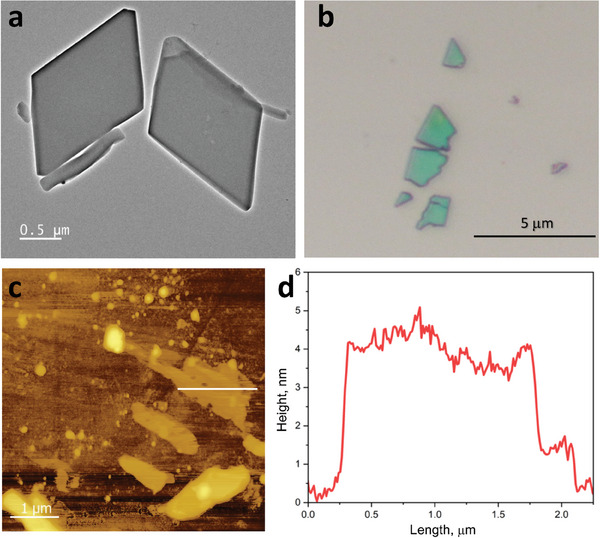
a) TEM micrograph of **2**. b) Exfoliated nanosheets **2** by a mechanical way.^[^ ^6a^ ^]^ c,d) AFM analysis of the exfoliated nanosheets of **2** with 4 nm thick (corresponding to app. 6 layers from Figure 1e).

Additionally, transmission electron microscopy (TEM) has been utilized to characterize the obtained **2** (**Figure** **3**a; Figure S19, Supporting Information). Being very sensitive materials to an electron beam, MOFs require low electron dose regime (lower than 100 e^–^/Å^2^)^[^ ^13g–j^ ^]^ for TEM analysis. Thus, we set the electron dose at 14.6 e^–^/Å^2^ per second using a Titan ST field‐emission electron microscope (Thermo‐Fisher Scientific) equipped with OneView camera (Gatan). It has been found that **2** possessed a rhombic shape (Figure 3a). However, upon low electron dose imaging, **2** amorphized very fast (Figure S19, Supporting Information), confirming the fact of the presence of weak interactions in **1** and **2**, driven the 3D to 2D transformation.

Next, assuming that the transformation of **1** to **2** is correlated with the 3D→2D process, the latter should cause the flaking and could be easily demonstrated by mechanical exfoliation.^[^ ^6a^ ^]^ Thus, the as‐synthesized crystals of **1** have been prepared for the mechanical exfoliation: **1** dried on a heating plate at 40 °C for 10 min (**1′**); and **1** dried on a heating plate at 50 °C for 5 h (**2**). As one can see in Figure S20 (Supporting Information), **1′** demonstrated an inability to be exfoliated due to maintained 3D structure (Figure 2a). In contrast, we have easily exfoliated **2**: The optical (Figure 3b) and atomic force microscopy (AFM, Figure 3c,d) revealed the flat and homogeneous nanosheets with the thickness of up to 4 nm (corresponding to app. 6 layers of **2**, Figure 1e).

The above results concerning the transformation of **1** during washing, drying, or heating allowed us to speculate about the 3D→2D structural transformation of MOF, driven by impairing the coordination of less favorable Zn‐N bonds between Zn clusters and BPE ligands. This has been also supported by the density functional theory (DFT) calculation, revealing an energy benefit of the process of temperature‐induced BPE ligand detachment followed by the coordination of H_2_O (or DMF) molecules with the resulting 2D structure (see Table S7, Supporting Information). Nevertheless, the Raman spectroscopy showed that the BPE ligand remained partially coordinated with the 2D framework, forming defects useful for electronic applications (see below).^[^ ^13k^ ^]^

**Figure 4:**
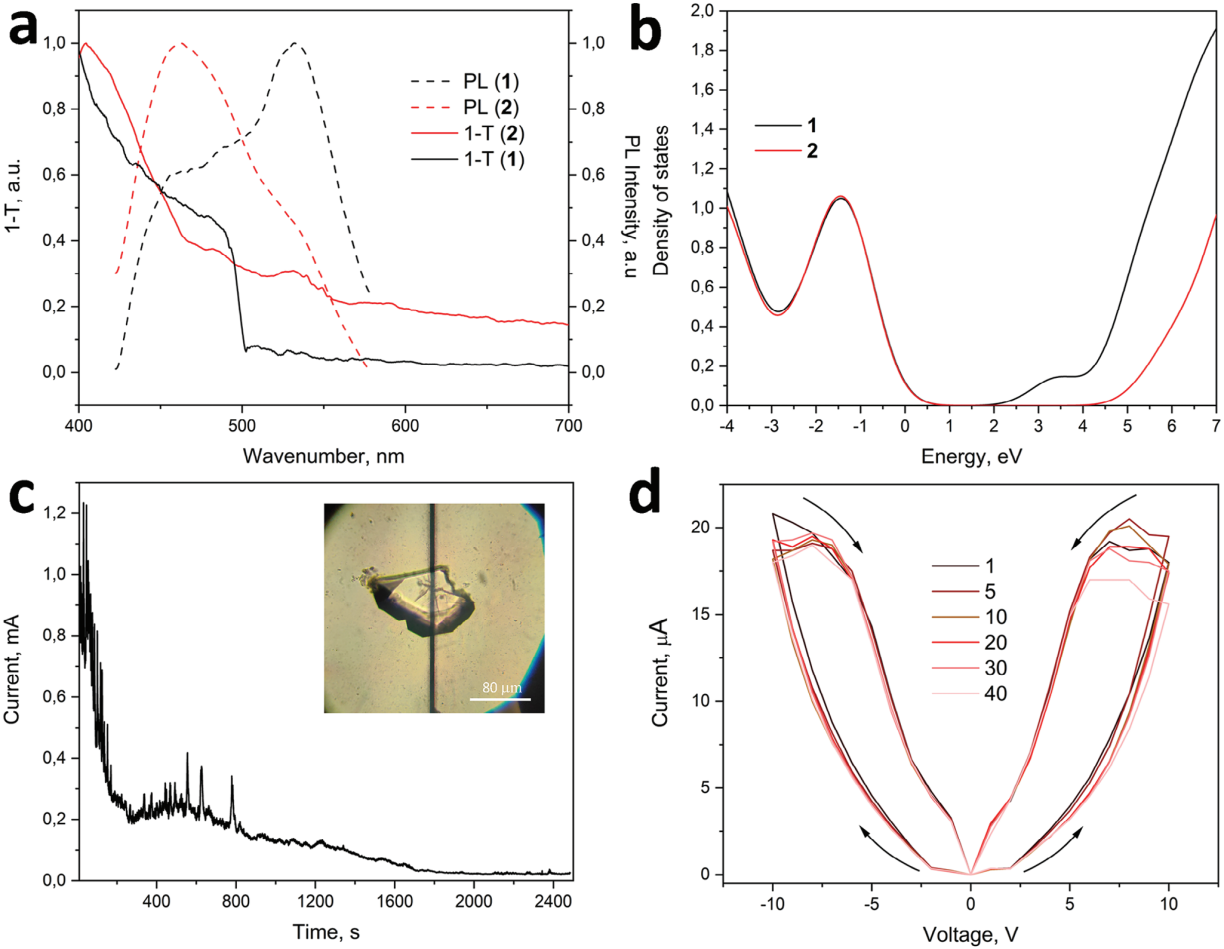
a) The optical absorption (1‐*T*, where *T* denotes the transmittance spectra, for more details see Experimental section) and photoluminescence (PL) spectra of single crystals of **1** and **2**. b) Total density‐of‐states (DOS) for **1** and modeled **2** with two coordinated H_2_O molecules (see also Figure S14, Supporting Information). c) 50‐fold decrease of the current passing through **1** during 40 min of the structural transformation to **2**. Inset: single crystal of **1** placed between two Au electrodes with 8 µm groove. d) The current‐voltage curves of switching from the high resistive to the low resistive states of bulk crystal of **2** over 40 cycles. Arrows indicate the set/reset path.

Generally, the change in the dimensionality of the crystal causes significant changes in its electronic properties, thus we have analyzed the optical absorption and PL spectra of single crystals **1** and **2** (see Experimental section). As can be seen in **Figure** **4**a, the transformation from **1** to **2** has been accompanied by a 0.2 eV blueshift of the absorption band (Figures S11 and S12, Supporting Information) and the appearance of a broad Urbach tail due to diffusion scattering of light by the nanosheets. Tauc plots (Figure S12, Supporting Information), reconstructed from Figure 4a, showed the band gap of 2.43 ± 0.04 eV and 2.65 ± 0.05 eV for **1** and **2**, respectively. The data for **2** has been also verified by diffuse reflectance UV–vis spectoroscopy (DRS UV‐vis), revealing the bandgap value of 2.75 ± 0.05 eV (Figure S13, Supporting Information). This spectral shift between **1** and **2** has been also confirmed qualitatively by DFT: Figure 4b and Figure S14 (Supporting Information) show app. 1 eV (depending on the coordinated H_2_O or DMF molecules with the resulting 2D structure) blue shit of the lowest unoccupied molecular orbital (LUMO) energy for the modeled structure of **2**, compared with that of **1**. Moreover, the changes in the optical absorption spectra led to a significant modification of the PL shape (Figure 4a; Figure S11, Supporting Information) and corresponding 100 nm blue shift of PL peak for **2**.

Next, we have tested the changes in resistive properties of MOF single crystals during the 3D→2D transformation (see Experimental section). For this, we have made a prototype of resistive switching element (Figure S15, Supporting Information), based on a single crystal of **1**, placed in a 8 µm groove between 100 nm Au contacts on a glass substrate (see Experimental section and inset in Figure 4c). As can be seen in Figure 4c, keeping of **1** at ambient conditions for 40 min led to a change in its color (Figure 2c,d) and a 50‐fold decrease of the current passing through the MOF. The latter corresponds to the transformation of **1** to a more insulating state, overlapping with 3D→2D structural transformation. For the resulting state (i.e., **2**), we have then performed current‐voltage and current AFM analysis. As one can see in Figure 4d, the current‐voltage curves can be recorded over 40 cycles of switching from the high resistive (HRS) to the low resistive states (LRS) of bulk crystal of **2**. Taking into account the instrument error during the current‐voltage measurements, the resulting 40 curves overlap perfectly, proving the memristive behavior of **2** with ON/OFF ratio of 6 at V_read_ = 5 V and V_set_ = 10 V (corresponding to 0.6 and 1.2 V µm^−1^ electric field strength, respectively).^[^ ^14a,b^ ^]^

**Figure 5:**
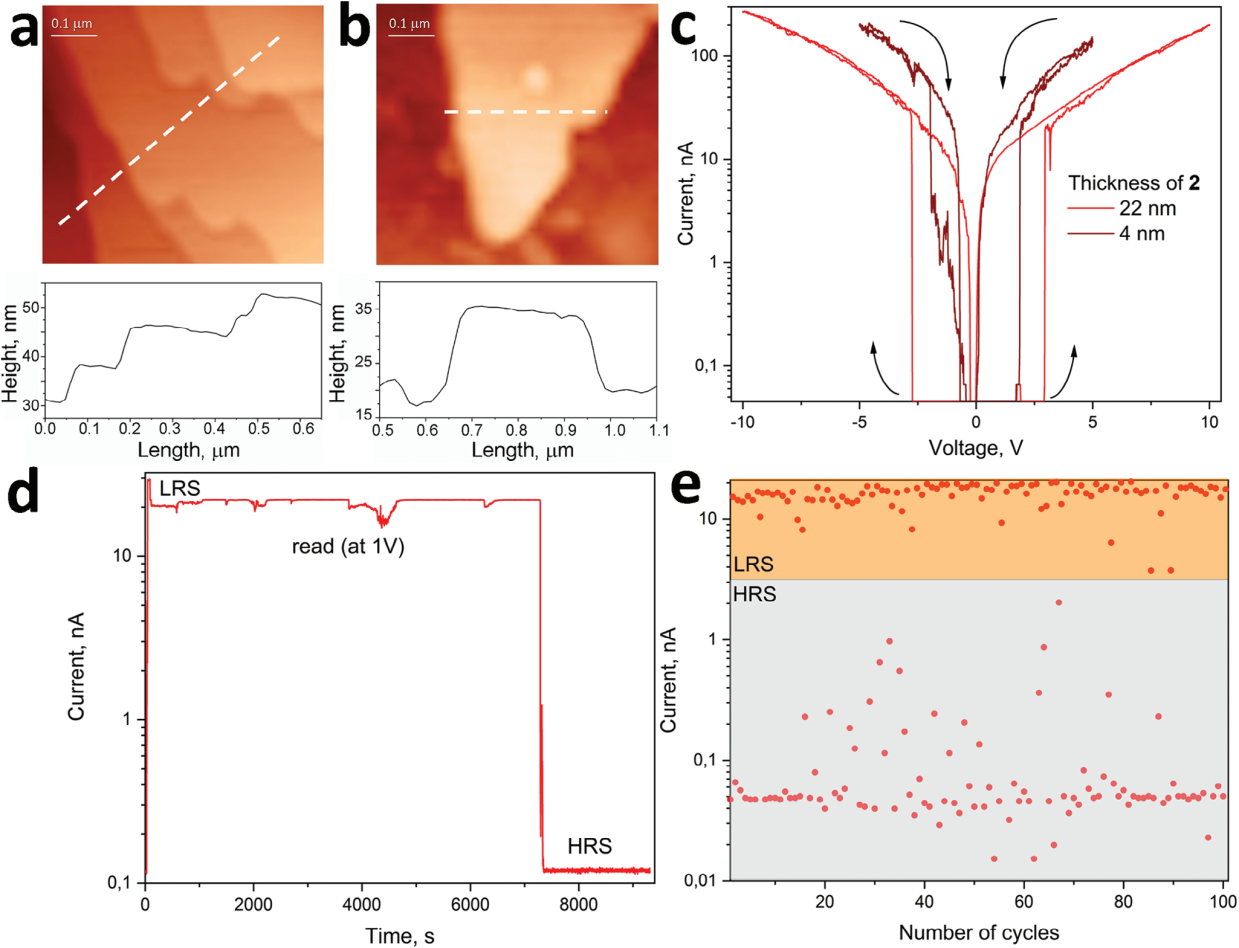
a,b) The topography of the nanosheets of Zn‐NDC/BPE (**2**) with stepped layered morphology captured during the AFM measurement: 5 to 11 nm (a); and 17 nm (b). c) Current–voltage curve of switching the resistance from high to low resistive states for layers of **2** with thickness of 4 nm (brown curve) and 22 nm (red curve). Arrows indicate the set/reset path. d) Retention time for the low resistive state, written by conductive AFM tip at 9 V applied voltage and read at 1 V during 9400 s. The nanosheet of **2** thickness was 32 nm. e) Endurance (number of switching cycles) for the nanosheets of Zn‐NDC/BPE (**2**) with the thickness of 22 nm.

Since the established memristive parameters for bulk crystal of **2** cannot be considered as a desirable value for MOFs in general,^[^ ^14a,b^ ^]^ we focused then on current‐AFM test of ultrathin nanosheets of **2** (4 to 32 nm, **Figure** **5**a,b) obtained by mechanical exfoliation on gold substrate. First, it has been found that the switching time of **2** to its LRS state depends significantly on the thickness: For instance, a 22 nm nanosheet has been switches to LRS at an applied voltage of 10 V with 1 V s^−1^ ramp rate, while the thicker samples could not be converted to the LRS when this voltage has been applied. Second, we have detected 150 to 1400 ON/OFF ratio for the nanosheets of **2** of a varied thickness (Figure 5c; Figure S18b,c, Supporting Information, at V_read_ = 1.5 V), being already quite competitive with MOF‐based memristive devices.^[^ ^14a,b^ ^]^ Moreover, the analysis of the dependence of the set voltage (Figure S18a, Supporting Information) on the nanosheet thickness and the ramp rate (V s^−1^) indicated a linear growth of V_set_ from 1.8 to 4.5 V for 4 to 32 nm thick nanosheets, as expected for insulating nanosheets.^[^ ^14c^ ^]^ Herein, nonlinear growth of set voltage on the ramp rate (Figure S18d, Supporting Information) allowed us to speculate about possible mechanisms of memristive behavior of **2**: Given the complex hierarchical structure of MOFs, such mechanisms as defect migration, phase transition, vacancy migration, filament formation, charge tunneling, and charge trapping can be simultaneously observed and compete inside the MOF.^[^ ^14g^ ^]^ Following the results of Figure S18d (Supporting Information), we have revealed a nonlinear shape of the dependence of V_set_ on *log*(ramp rate), which may be a confirmation of the complexity^[^ ^14h,i^ ^]^ of the memristive processes in **2**. With a greater probability, the defect migration and charge trapping (by the residual BPE ligand), including the conventional filament formation could occur inside porous MOF structure. Next, we have measured the retention time (Figure 5d) and the number of switching cycles for the nanosheets of **2** (Figure 5e), whose values (7300 s and 100 cycles, respectively) are comparable with similar MOF‐based prototypes.^[^ ^14d^ ^]^ Also, data on the amplitude of current, nanosheet thickness, and the parameters of conductive AFM tip (see Experimental section) allowed us to estimate the conductivity of **2**. It turned out that the conductivity was app. 10^−4^ S cm^−1^ in the low‐resistive state (see Supplementary Information), being higher than that for most MOFs in 3D and 2D form.^[^ ^14e^ ^]^ However, such value is still inferior to the conductivity of ultrathin 2D MOFs and COFs with extremely small band gaps.^[^ ^14f^ ^]^ Despite this, the proposed concept of the transformation of 3D MOFs into 2D form through direct synthesis followed by thermal/solvent treatment makes it possible to technologically obtain a large number of high‐quality and homogeneous MOF nanosheets; the high temperature stability of which (up to 400 °C, see thermogravimetric analysis, TGA, in Figure S3, Supporting Information) and the presence of specific defects (like residual BPE ligand) opens up a new perspectives of scalable and insulating 2D MOFs for micro‐ and opto‐electronic applications.

## 3 Conclusion

We have demonstrated the concept of 3D to 2D MOFs structural transformation through the utilization of neutral N‐donor ligands. Initially, we designed and solvothermally synthesized 3D MOFs (Co‐ and Zn‐NDC/BPE) based on 1,2‐bis(4‐pyridyl)ethylene (BPE) and 2,6‐naphthalenedicarboxylate (NDC) ligands, followed by their thermal and solvent treatment to implement the transformation. A set of single crystal and powder X‐ray diffraction, electron microscopy, Raman spectroscopy, numerical modeling, and mechanical exfoliation confirmed the nature of MOF transformation. Compared with the initial 3D MOF, its nanosheets demonstrated sufficient changes in electronic properties, expressed as tuning their optical absorption (by a 0.2 eV blue shift), PL (by a 100 nm blue shift), and resistivity by 50 times. The latter allowed us to demonstrate the prototype of ultrathin memristive element based on a 4 to 32 nm MOF nanosheet with enhanced functionality (150 to 1400 ON/OFF ratio, retention time exceeding 7300 s, and 100 cycles of switching), thereby, extending the list of scalable and insulating 2D MOFs for micro‐ and opto‐electronics.

## 4 Experimental Section

### Materials

Zn(NO_3_)_2_·6H_2_O (Sigma‐Aldrich, 98%), Co(NO_3_)_2_·6H_2_O (Sigma‐Aldrich, 98%), 2,6‐Naphthalenedicarboxylic acid (Sigma‐Aldrich, 95%), 1,2‐Bis(4‐pyridyl)ethylene (Sigma‐Aldrich 97%), dimethylformamide (ACS reagent, 99.8%).

### MOF Synthesis

Zn‐NDC/BPE (CCDC 2347223) single‐crystals were synthesized using a solvothermal method. For this, Zn(NO_3_)_2_·6H_2_O (0.125 mmol), 1,2‐Bis(4‐pyridyl)ethylene (0.25 mmol), and 2,6‐Naphthalenedicarboxylic acid (0.25 mmol) were dissolved in 12.5 ml of DMF separately. 1 ml of each ligands were added to 2 ml of Zn(NO_3_)_2_·6H_2_O by sliding down the walls of the 4 ml vial. Then, the vial with a final solution was left in an oven for 48 h at 80 °C. The obtained microcrystals of Zn‐NDC/BPE were kept in a mother solution (DMF) afterward to avoid degradation after the washing process. The synthesis of Co‐NDC/BPE (CCDC 2347222) was similar to Zn‐NDC/BPE.

### SCXRD

Single crystal X‐ray diffraction was performed on a Bruker APEX‐II CCD diffractometer. The crystal was kept at 297.57 K during data collection. Using Olex2, the structure was solved with the SHELXT structure solution program using Intrinsic Phasing and refined with the SHELXL refinement package using Least Squares minimization (Tables S2 and S3, Supporting Information).^[^ ^15a,b^ ^]^

### PXRD

Powder X‐ray diffraction patterns were obtained on a Shimadzu XRD 7000S powder diffractometer (Cu‐Kα radiation, *λ* = 1.5406 Å). Data have been collected in the range of 5–40° (Figure 2a,b; Figure S5, Supporting Information).

### SEM

Scanning electron microscope FEI Quanta 200 FEG MKII with the Oxford Inca Energy Dispersive X‐ray (EDX) system was used for the analysis of morphology of obtained crystals and performing the structural transformation dynamics of crystals upon electronic beam, operating at 20 kV (Figure 2e; Figures S6–S8, Supporting Information).

### Raman Spectroscopy

The confocal Raman measurements were performed on the confocal microscope setup. The single crystals of MOFs were irradiated by coherent light (He−Ne source with a wavelength of 632.8 nm, 15 mW) via a 100x/0.9NA objective. The scattered light was collected via the same objective and then analyzed using the confocal Raman spectroscopy system HORIBA Labram with 1800 g mm^−1^ diffraction gratings and water‐cooling camera ANDOR (Figure 2f; Figures S16 and S17, Supporting Information).

### Optical Transmittance

To qualitatively evaluate the shape of an optical absorption spectra of MOF single crystals (*A*, Figure 4a; Figure S12, Supporting Information), transmission spectroscopy, *T* (*A* = 1 – *T*, taking into account the insignificant reflection and diffusion scattering values) has been implemented. For this, single crystals of **1** and **2** have been irradiated with a white halogen lamp (360–2500 nm, Avantes). The white light was focused with an Mitutoyo 10 × /0.28 NA objective and the transmitted signal was collected using an Mitutoyo 50 × /0.42 NA objective. HORIBA LabRAM confocal spectrometer with a water‐cooled charge‐coupled device (CCD, Andor DU 420A‐OE 325), and 150 g mm^−1^ diffraction grating were used to analyze the spectra.

### Photoluminescence

The PL measurements have been performed on the same setup as for transmittance spectroscopy. The single crystals of **1** and **2** were irradiated by coherent light (Yb^3+^ femtosecond laser source with a wavelength of 350 nm, 150 fs pulse duration, 80 MHz repetition rate) via a 100x/0.9NA objective. The re‐emitted light was collected via the same objective and then analyzed using the confocal spectroscopy system HORIBA Labram with 150 g mm^−1^ diffraction gratings and water‐cooling camera ANDOR (Figure 4a).

### DFT

The density functional theory calculations for **1** and **2** (Figure 4b; Figure S14 and Table S7, Supporting Information) based on the experimentally obtained single crystal X‐ray structure of **1** has been carried out using the dispersion‐corrected hybrid functional ωB97XD with the help of Gaussian‐09 program package.^[^ ^15c,d^ ^]^ The 6–31+G* basis sets were used for all atoms. The Multiwfn program (version 3.7) was used for total density‐of‐states (TDOS) calculations and topological analysis of the election density distribution.^[^ ^15e,f^ ^]^ The frontier molecular orbitals were visualized in Chemcraft program.

### Mechanical Exfoliation

As‐synthesized Zn‐NDC‐BPE MOF crystals (**1**) were heated on a substrate at a temperature of 40 °C for 10 minutes for total solvent (DMF) evaporation. After that, Nitto blue tape was utilized for mechanical exfoliation as described in details in ref. [6a]. The first piece of the tape fixed single crystals and the second pieced stamped upon the first for the split of MOF. After several iterations of stamping, the tape was glued to a silicon wafer (or gold substrate for current‐AFM), pressed, and slowly removed (Figure 3).

### Current–Voltage Characterization

Single Zn‐NDC‐BPE (**1**) crystals were placed on a glass substrate coated with 100 nm of Au and 5 nm of Cr sublayer, deposited using the thermal evaporation method. Using the laser ablation method, an 8 µm wide groove was made followed by MOF deposition on it. For a detailed analysis of the resistive switching, a home‐made setup enabling automatic data recording was used (Figure S15, Supporting Information): Keithley 2700 multimeter (10 nA sensitivity) and an Element 305 dB power supply were used. Automation was achieved through the controller allowing adjustment of the power source voltage, polarity, and data retrieval from the multimeter. The current–voltage curves were recorded immediately in air after laying the crystal **1** on the contacts. The analysis of current‐voltage parameters has been performed with applying a cyclic potential of −10 to 0 to 10V with a step of 1V, compliance of 0.1A, and a pause of 1s.

### Conductive AFM

Measurements of conductivity maps I(x,y) for **2**, current–voltage characteristics I(V), and time dependences I(t) were carried out using the method of conductive contact atomic force microscopy with an NTegra‐Aura microscope (NTMDT, Russia), and HA_C/W2C probes (TipsNano, Russia) with a conductive wear‐resistant W_2_C coating (Figure 5).

### BET Surface Area

Nitrogen sorption−desorption data along with surface area analysis, pore sizes, and pore volume were analyzed with the Brunauer−Emmett−Teller surface area and pore size analyzer Quantachrome NOVA 1200 e. Degassing of samples was performed at room temperature for 40 h for 3D Zn‐NDC/BPE, and at 100 °C for 24 h for 2D Zn‐NDC/BPE (Table S5, Supporting Information).

### XPS

X‐Ray photoelectron spectroscopy analysis was performed on Thermo Fisher Scientific Escalab 250Xi spectrometer with Al Kα radiation as the excitation source (Figures S9 and S10, Supporting Information).

### TGA

Thermogravimetric analysis was performed using TGA 92‐16.18 by from SETARAM under argon flow at a heating rate of 3 °C min^−1^ until 605 °C (Figure S3, Supporting Information).

### DRS UV–vis

Diffuse Reflectance UV–vis spectoroscopy (DRS UV–vis) was performed by Shimadzu UV‐2550 for the absorbance, reflectance, and bandgap determination. The spectra were obtained for 2D Zn‐NDC/BPE (**2**) in a barium sulfate matrix. The spectra from a pure barium sulfate matrix was subtracted from the resulting spectra. For the quantitative description of the diffuse scattering spectra, the basis of the Kubelka‐Munk theory was applied. The absorbance and reflectance Tauc plots were reconstructed, and indirect bandgap was calculated.

### TEM

TEM analysis has been carried out with a Titan ST field‐emission electron microscope (Thermo‐Fisher Scientific) equipped with OneView camera (Gatan). The particles were dispersed on a carbon‐copper grid. Analysis was performed with an accelerating voltage of 300 kV at Gun Lens set to 8.

## Conflict of Interest

The authors declare no conflict of interest.

## Supporting information



Supporting Information

Supplemental Video 1

## Data Availability

The data that support the findings of this study are available in the supplementary material of this article.
