# Correction to “Transformation of 3D Metal–Organic Frameworks into Nanosheets with Enhanced Memristive Behavior for Electronic Data Processing”

**DOI:** 10.1002/advs.202514348

**Published:** 2025-09-12

Yuri A. Mezenov, Semyon V. Bachinin, Yuliya A. Kenzhebayeva, Anastasiia S. Efimova, Pavel V. Alekseevskiy, Daria Poloneeva, Anastasia Lubimova, Svyatoslav A. Povarov, Vladimir Shirobokov, Mikhail S. Dunaevskiy, Aleksandra S. Falchevskaya, Andrei S. Potapov, Alexander Novikov, Artem A. Selyutin, Pascal Boulet, Jorge Gascon, Alena N. Kulakova, Valentin A. Milichko, Advanced Science 12, 2405989 (2025)

https://doi.org/10.1002/advs.202405989

Prof. Jorge Gascon (King Abdullah University of Science and Technology, KAUST Catalysis Center (KCC), Thuwal 23955‐6900, Saudi Arabia), as a supervisor of our co‐author and a grant holder, covering the HRTEM analysis at KAUST, was missed unintentionally from the list of authors.

We apologize for this error.

