# Supplementary material for: Transformation of 3D Metal–Organic Frameworks into Nanosheets with Enhanced Memristive Behavior for Electronic Data Processing
Source: Adv Sci (Weinh). 2025 Mar 2;12(16):2405989. doi: 10.1002/advs.202405989 (PMC12021068; doi:10.1002/advs.202405989)
Supplement: Supplementary file 1 — Supporting Information [file ADVS-12-2405989-s001.docx]

Supporting Information

Transformation of three-dimensional metal-organic frameworks into nanosheets with enhanced memristive behavior for electronic data processing

Yuri A. Mezenov,^†^ Semyon V. Bachinin, ^†^ Yuliya A. Kenzhebayeva, Anastasiia S. Efimova, Pavel V. Alekseevskiy, Daria Poloneeva, Anastasia Lubimova, Svyatoslav A. Povarov, Vladimir Shirobokov, Mikhail S. Dunaevskiy, Aleksandra S. Falchevskaya, Andrei S. Potapov, Alexander Novikov, Artem A. Selyutin, Pascal Boulet, Alena N. Kulakova,* Valentin A. Milichko*

Y. A. Mezenov

Qingdao Innovation and Development Center, Harbin Engineering University, Qingdao 266000, Shandong, China

Y. A. Kenzhebayeva, A. S. Efimova, P. V. Alekseevskiy, A. Lubimova, S. V. Bachinin, S. A. Povarov, V. Shirobokov, M. S. Dunaevskiy A. N. Kulakova, V. A. Milichko

School of Physics and Engineering, ITMO University, St. Petersburg, 197101, Russia.

E-mail: v.milichko@metalab.ifmo.ru; alena.kulakova@metalab.ifmo.ru

D. Poloneeva

Advanced Catalytic Materials (ACM), KAUST Catalysis Center (KCC), Division of Physical Sciences and Engineering, King Abdullah University of Science and Technology, Thuwal, 23955 Saudi Arabia

Aleksandra S. Falchevskaya

ITMO University, “Solution Chemistry of Advanced Materials and Technologies” (SCAMT) International Institute, Saint Petersburg 191002, Russia

A. S. Potapov

Nikolaev Institute of Inorganic Chemistry Siberian Branch of the Russian Academy of Sciences, Laboratory of metal-organic coordination polymers, Novosibirsk, 630090, Russia

A. Novikov, A. A. Selyutin

Saint Petersburg State University, Saint Petersburg, 199034, Russia

A. Novikov

Рeoples’ Friendship University of Russia, Moscow, 117198, Russia

P. Boulet, V. A. Milichko

Institut Jean Lamour, Universit de Lorraine, UMR CNRS 7198, 54011 Nancy, France

^†^ The authors contributed equally

**Table S1.** Reported structural transformations of MOFs with their dimensionality changes.

| MOF | Transformation | Method | Ref. |
| --- | --- | --- | --- |
| [Cu(bpp)_2_(BF_4_)_2_] | 3D→1D | Liquid/vapor treatments | [S1] |
| Mo_2_(INA)_4_ | 1D→2D | Vapor treatment | [S2] |
| [Zn_2_L(DMF)_4_]⋅2DMF⋅4H_2_O | 2D→3D | Guest induced | [S3] |
| {[Cu(μ-atrz)_3_]·(BF_4_)_2_·2H_2_O}_n_ | 3D→2D | Anion induced | [S4] |
| Cu-THQ | 2D→3D | Post-syntheric pillard insertion | [S5] |
| COK-18 | 1D→2D→3D | Thermal treatment | [S6] |
| [Cd(IBA)_2_]_n_ | 2D→3D | Thermal treatment | [S7] |
| [Zn_3_(BPDC)_3_(BiPY)]⋅solvent | 3D→2D | Ligand exhancge | [S8] |
| poly(arylenevinylene) | 2D↔3D | Light-induced cycloaddition | [S9] |
| {[Cd(pzdc)(bpee)]_2_·3H_2_O}n | 3D→2D | Light-induced cycloaddition | [S10] |
| [Zn(4-spy)(DCTP)]n | 2D→3D | Light-induced cycloaddition | [S11] |
| MOF-5 | 2D↔3D | Variation of the ratio of precursors | [S12] |
| MSU-10, MSU-11 | 2D↔3D | Introducing a chemical modulator | [S13] |
| ([M_2_(bdc)(dabco)]·guest | 3D→2D | Ultrasonication | [S14] |
| [Co_2_·(μ3–O)·(Hnip)·(mtt)·(H_2_O)]·1.5H_2_O | 3D→2D | Solvent unduced | [S15] |
| Co_3_-MOF | 3D→2D | Ultrasonication | [S16] |

**Table S2.** Crystal data and structure refinement for Zn-NDC/BPE (**1**, CCDC 2347223).

| Empirical formula | C_13.5_H_7.75_N_0.75_O_3_Zn_0.75_ |
| --- | --- |
| Formula weight | 277.48 |
| Temperature/K | 297.57 |
| Crystal system | triclinic |
| Space group | P-1 |
| *a*/Å | 13.1235(13) |
| *b*/Å | 13.1906(12) |
| *c*/Å | 16.3393(16) |
| α/° | 97.642(3) |
| β/° | 94.866(3) |
| γ/° | 90.978(3) |
| Volume/Å^3^ | 2792.1(5) |
| Z | 8 |
| ρ_calc_g/cm^3^ | 1.320 |
| F(000) | 1124.0 |
| Crystal size/mm^3^ | 0.05 × 0.03 × 0.015 |
| Radiation | MoKα (λ = 0.71073) |
| 2Θ range for data collection/° | 3.116 to 55.584 |
| Index ranges | -17 ≤ h ≤ 17, -17 ≤ k ≤ 17, -21 ≤ l ≤ 21 |
| Reflections collected | 79101 |
| Independent reflections | 13127 [R_int_ = 0.0546, R_sigma_ = 0.0429] |
| Data/restraints/parameters | 13127/0/653 |
| Goodness-of-fit on F^2^ | 1.108 |
| Final R indexes [I>=2σ (I)] | R_1_ = 0.0422, wR_2_ = 0.1368 |
| Final R indexes [all data] | R_1_ = 0.0685, wR_2_ = 0.1491 |
| Largest diff. peak/hole / e Å^-3^ | 0.77/-0.51 |

**Table S3.** Crystal data and structure refinement for Co-NDC/BPE (CCDC 2347222).

| Empirical formula | C_12_H_8_Co_0.5_NO_2_ |
| --- | --- |
| Formula weight | 227.66 |
| Temperature/K | 297.57 |
| Crystal system | triclinic |
| Space group | P-1 |
| *a*/Å | 9.5281(8) |
| *b*/Å | 11.8733(10) |
| *c*/Å | 12.3642(11) |
| α/° | 78.472(3) |
| β/° | 68.619(3) |
| γ/° | 78.733(3) |
| Volume/Å^3^ | 1264.66(19) |
| Z | 4 |
| ρ_calc_g/cm^3^ | 1.196 |
| μ/mm^‑1^ | 0.706 |
| F(000) | 466.0 |
| Crystal size/mm^3^ | 0.06 × 0.03 × 0.02 |
| Radiation | MoKα (λ = 0.71073) |
| 2Θ range for data collection/° | 3.532 to 53.9 |
| Index ranges | -12 ≤ h ≤ 12, -15 ≤ k ≤ 15, -15 ≤ l ≤ 15 |
| Reflections collected | 26529 |
| Independent reflections | 5292 [R_int_ = 0.0474, R_sigma_ = 0.0477] |
| Data/restraints/parameters | 5292/0/281 |
| Goodness-of-fit on F^2^ | 1.041 |
| Final R indexes [I>=2σ (I)] | R_1_ = 0.0367, wR_2_ = 0.0961 |
| Final R indexes [all data] | R_1_ = 0.0521, wR_2_ = 0.1018 |
| Largest diff. peak/hole / e Å^-3^ | 0.29/-0.26 |

**Table S4.** 2D and 3D MOF structures of Zn-NDC alternatives.

| CSD Code | Formula | Synthesis conditions | Dimensionality | Ref. |
| --- | --- | --- | --- | --- |
| AGAXEL | [Zn(DMF)(ndc)] | Zn(NO_3_)_2_ + H_2_ndc, DMF/Et_3_N/PhCl, 72 h, r.t. | 2D | [S17] |
| AJOREY | {[Zn_2_(DMF)_2_(ndc)_2_]·2H_2_O} | Zn(NO_3_)_2_ + H_2_ndc, DMF/H_2_O, 48 h, 120 °C | 2D | [S18] |
| CIFFOO | [Zn_2_(DMF)_2_(ndc)_2_] | Zn(NO_3_)_2_ + H_2_ndc, DMF, 67 h, 120 °C | 2D | [S20] |
| EWEVAD | {[Zn_2_(DMF)_2_(ndc)_2_]·C_6_H_6_} | Zn(NO_3_)_2_ + H_2_ndc, DMF/Et_3_N/C_6_H_6_, r.t. | 2D | [S22] |
| EWEVEH | {[Zn_2_(DMF)_2_(ndc)_2_]·PhMe} | Zn(NO_3_)_2_ + H_2_ndc, DMF/Et_3_N/PhMe, r.t. | 2D | [S22] |
| EWEVIL | {[Zn_2_(DMF)_2_(ndc)_2_]·C_8_H_10_} | Zn(NO_3_)_2_ + H_2_ndc, DMF/Et_3_N/C_8_H_10_, r.t. | 2D | [S22] |
| ZULSIK | [Zn_2_(DEF)_2_(ndc)_2_] | Zn(OAc)_2_ + H_2_ndc, DEF/HCOOH, 17 h, 110 °C | 2D | [S33] |
| CAGSAG | [(Zn_4_O)_2_(DMF)_3_(H_2_O)(ndc)_6_] | Zn(NO_3_)_2_ + H_2_ndc, DMF, 24 h, 120 °C | 3D (2-fold) | [S19] |
| EDUTUS | [Zn_4_(ndc)_4_] | Zn(NO_3_)_2_ + H_2_ndc, DEF, 85-105 °C | 3D | [S21] |
| FECXUI | [(Zn_4_O)_2_(MeOH)(ndc)_6_] | Zn(NO_3_)_2_ + H_2_ndc, DMA/MeOH, 24 h, 100 °C | 3D (2-fold) | [S23] |
| FECYUJ | [(Zn_4_O)_2_(DMF)(H_2_O)_2_(ndc)_6_] | Zn(NO_3_)_2_ + H_2_ndc, DMF, 168 h, r.t. | 3D (2-fold) | [S23] |
| HAXCUI | {(Me_2_NH_2_)_4_[Zn_2_(ndc)_4_]} | Zn(NO_3_)_2_ + H_2_ndc, DMF, 24 h, 140 °C | 3D (3-fold) | [S24] |
| IDIXAU | [Zn_3_(OH)_2_(ndc)_2_] | Zn(NO_3_)_2_ + H_2_ndc, DEF/H_2_O_2_/MeNH_2_/DMF, 168 h, r.t. | 3D | [S25] |
| IYEXEP | [Zn_2_(OH)_2_(ndc)] | Zn(NO_3_)_2_ + H_2_ndc, Py/H_2_O, 24 h, 180 °C | 3D | [S26] |
| KODXIN | [Zn_4_(OH)_6_(ndc)] | Zn(OAc)_2_ + H_2_ndc, DMF | 3D | [S27] |
| MIMRAB | [Zn(H_2_O)(ndc)] | Zn(NO_3_)_2_ + H_2_ndc, H_2_O, 72 h, 180 °C | 3D | [S28] |
| PUWDEQ | {(Et_3_NH)_2_[Zn_2_(ndc)_3_]·PhCl} | Zn(NO_3_)_2_ + H_2_ndc, DEF/Et_3_N/PhCl, 168 h, r.t. | 3D | [S29] |
| QAQSAD | [Zn_3_(MeOH)_2_(ndc)_3_] | Zn(NO_3_)_2_ + H_2_ndc, DMF/MeOH, 16 h, 60 °C | 3D | [S30] |
| USAHAZ | {(Et_2_NH_2_)[Zn_2_(OAc)(ndc)_2_]} | Zn(NO_3_)_2_ + H_2_ndc, DEF/AcOH, 5 h, 130 °C | 3D | [S31] |
| WORKOF | [(Zn_4_O)_4_(DMF)_4_(H_2_O)_4_(ndc)_12_] | Zn(NO_3_)_2_ + H_2_ndc, DMF/dioxane, 24 h, 110 °C | 3D (2-fold) | [S32] |
| WORKUL | [(Zn_4_O)_2_(H_2_O)_4_(ndc)_6_] | transformation in EtOH, 72 h | 3D (2-fold) | [S32] |
| WORLAS | [(Zn_4_O)_2_(DMF)_3_(H_2_O)(ndc)_6_] | Zn(NO_3_)_2_ + H_2_ndc, DMF/dioxane, 24 h, 110 °C | 3D (2-fold) | [S32] |
| BIHMEO | {[Zn_2_(bpe)(ndc)_2_]·xG} | ZnCl_2_ + H_2_ndc, DMF/H_2_O, 72 h, 90 °C | 3D (3-fold) | [S34] |
| RELLAW | [Zn_2_(bpe)(ndc)_2_] | Zn(NO_3_)_2_ + H_2_ndc, DMF, 24 h, 100 °C | 3D (2-fold) | [S35] |

**Table S5**. Brunauer-Emmett-Teller (BET) experimental surface area analysis of 2D Zn-NDC/BPE MOFs (**2**) and calculated value for 3D Zn-NDC/BPE MOFs (**1**) by PoreAnalyzer function, Mercury.

| MOF | BET surface area, m^2^ g^-1^ |
| --- | --- |
| Zn-NDC/BPE (**1**), modelled | 79 |
| Zn-NDC/BPE (**2**) | 236 |

The porous structure was analyzed using the nitrogen adsorption technique on a Quantachrome’s Autosorb iQ gas sorption analyzer at 77 K. Initially, the compound was activated under a dynamic vacuum at 80 °C for 8 h. The nitrogen adsorption−desorption isotherms were measured within the range of relative pressures from 10^−6^ to 0.995. The specific surface area was calculated from the data obtained using the conventional BET, Langmuir, and DFT models. The adsorption isotherm was fitted to a linear form in the relative pressure range P/P_0_ (1·10^–3^, 0.2)

| $\frac{1}{n\left( \frac{P_{0}}{P}-1 \right)}=\frac{1}{wC}+\frac{C-1}{wC}\frac{P}{P_{0}}$ | (1) |
| --- | --- |

*n* — uptake in mmol·g^–1^, *P*/*P*_0_ — relative pressure, *w* — monolayer capacity in mmol·g^–1^, *C* — BET constant.

BET surface area was calculated using the equation:

| $S_{BET}=w\cdot a_{m}\cdot N_{A}$ | (2) |
| --- | --- |

*a_m_* = 16.2 Å^2^, nitrogen cross-section area, *N_A_* — Avogadro number.

**Figure S1.** Nitrogen adsorption-desorption isotherms at 77 K (a) and BET plot (b) for MOF **2**.

**Table S6.** The parameters of porous structure of **2** under investigation.

| **Specific surface area / m^2^·g^−1^** | | | ***V*_pore_ / cm^3^·g^−1^** | | ***V*_ads_(N_2_)***^а^***/ cm^3^(STP)·g^−1^** |
| --- | --- | --- | --- | --- | --- |
| **Langmuir** | **BET** | **DFT** | Total*^a^* | DFT |  |
| 364 | 236 | 225 | 0.144 | 0.149 | 93.0 |

*^a^* measured at *P*/*P*_0_ = 0.95.

Scheme S1. Schematic representation of the transformation process of 3D MOF into 2D nanosheets via the neutral monodentate ligands of BPE driven by the external stimuli.


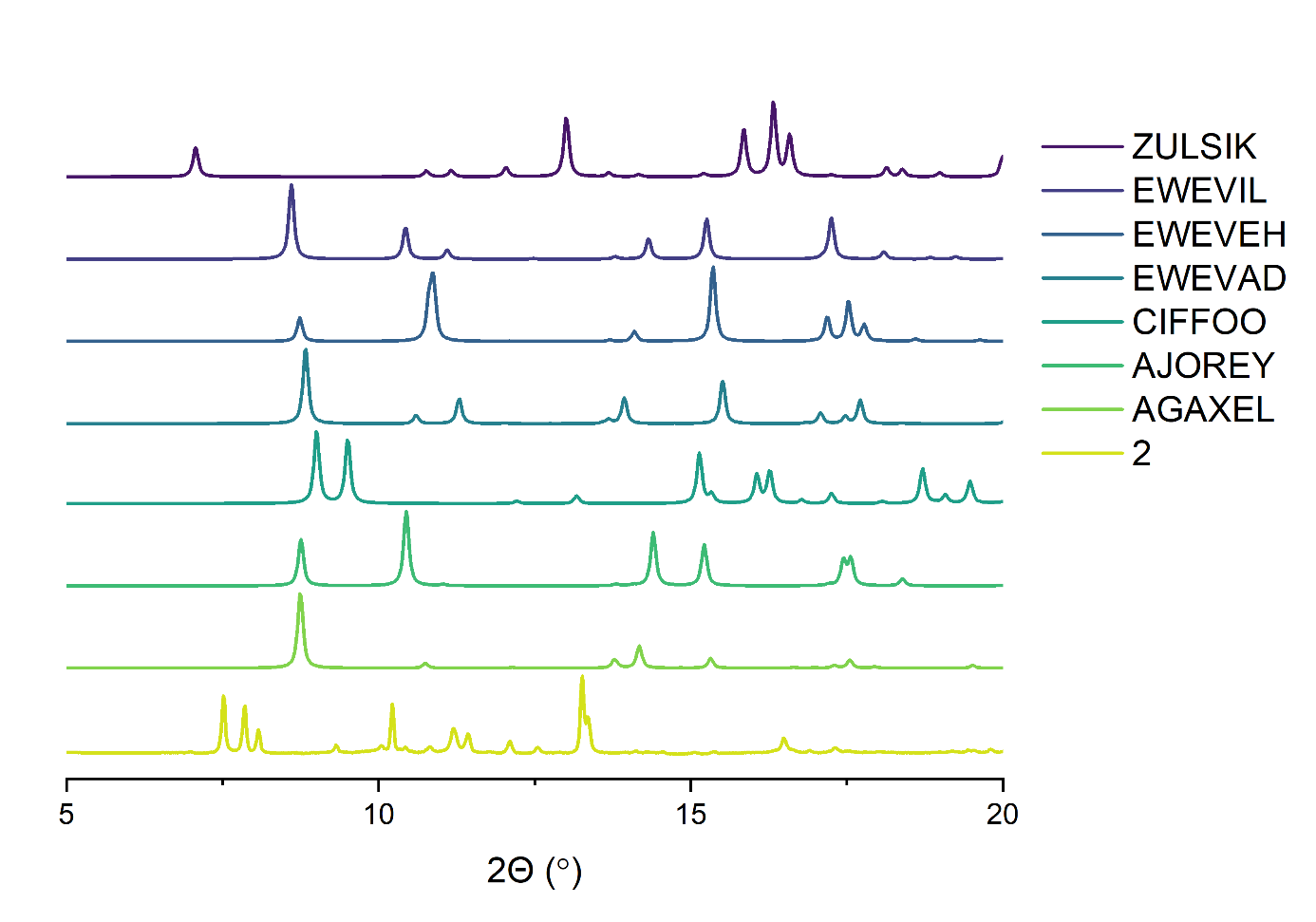


**Figure S2.** A comparison of the PXRD patterns of the obtained 2D MOF Zn-NDC/BPE (**2**) with other 2D structures based on Zinc and NDC ligand (with corresponding CSD code, see also Table S4).

**Figure S3.** TGA analysis of the 2D Zn-NDC/BPE **2** (a) and 2D Co-NDC/BPE (b).


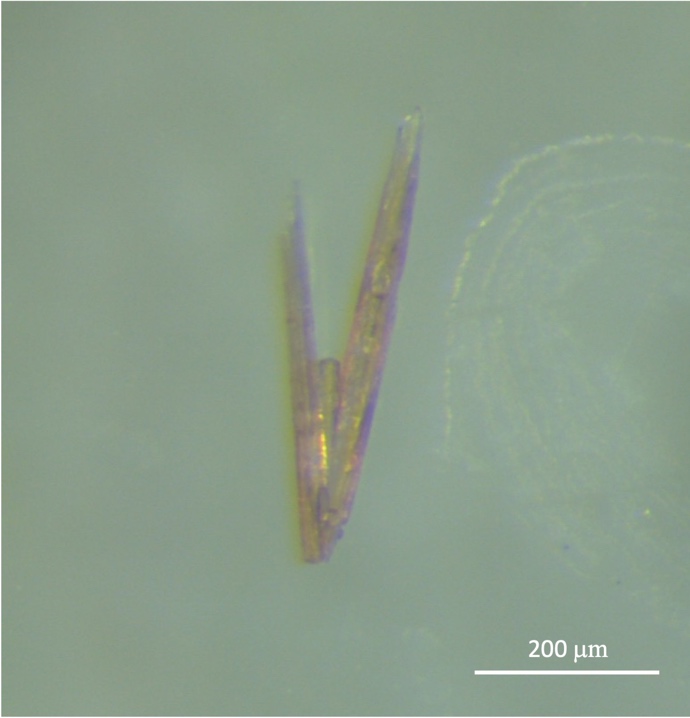


**Figure S4.** Optical image of crystals of Co-NDC/BPE.





**Figure S5.** PXRD pattern of the as-synthesized 3D Co-NDC/BPE (see also Table S3).

**Figure S6.** SEM micrographs of the heated single crystals of **1** (for transformation to **2**) at 80 °C (a-d), 100 °C (e-h), and 120 °C (i-l) on the heating plate in air.

**Figure S7.** SEM micrographs of the dried crystals of **1** in vacuum.

**Figure S8.** SEM micrographs of the layered structure of 2D Zn-NDC/BPE (**2**).

**Figure S9.** XPS analysis of the full region of 3D Zn-NDC/BPE **1** (a) and different elements Zn 2p (b), O 1s (c), N 1s (d), C 1s (e).

**Figure S10.** XPS analysis of the full region of 2D Zn-NDC/BPE **2** (a) and different elements Zn 2p (b), O 1s (c), N 1s (d), C 1s (e).

X-ray photoelectron spectroscopy (XPS) was used to verify the composition and purity of the 3D Zn-NDC/BPE (**1**) and 2D Zn-NDC/BPE (**2**). Figures S9a, S10a display the survey spectra of the existence of major elements such as Zn, O, N, C. The binding energy of Zn 2p spectra for both **1** and **2** revealed two peaks of Zn^2+^ at ~1044.3 eV and ~1021.3 eV corresponding to Zn 2p1/2 and Zn 2p3/2, respectively (Figures S9b, Figures S10b).^S49^ This demonstrates that the charge on the metal didn’t change due to structural transformation from 3D to 2D. The O 1s region of **1** and **2** were split into two peaks: Zn-O bonds at ~530.6 eV and a peak at ~532.4 eV of C=O bonds or surface adsorbed oxygen from water molecules (Figures S9c, Figures S10c). Importantly, the full width at half maximum (FWHM) values of O 1s region for **1** and **2** (1.86 and 1.97 eV, respectively) increased after the 3D to 2D transformation, what can indicate the increase of absorbed H_2_O after the transformation.^S50,S51^ The N 1s peaks are located at ~400.5 and 398.9 eV for **1**, and at ~400.7 and 398.9 eV for **2** (Figures S9d, Figures S10d). The XPS signal of C 1s for both **1** and **2** allocated at ~287.3 and ~284.0 eV and attributed to the C-O/C=O and C-C bonds, respectively (Figures S9e, S10e).^S49^


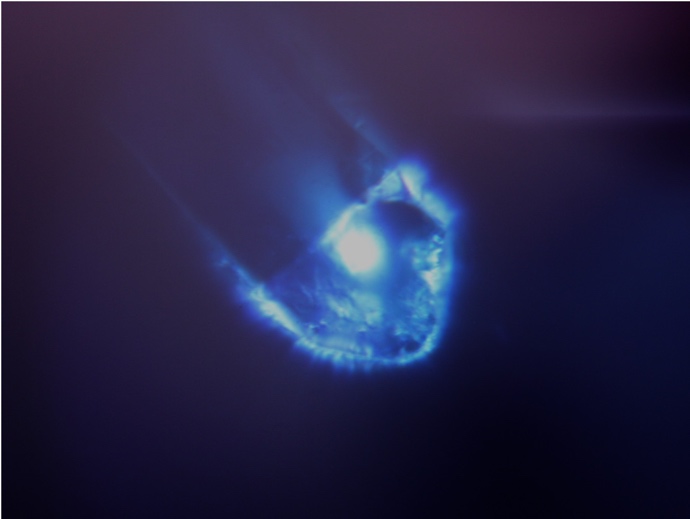


**Figure S11.** Optical image of PL from Zn-NDC/BPE (**2**), initiated by upon Yb^3+^ femtosecond laser with a wavelength of 350 nm, 150 fs pulse duration, and 80 MHz repetition rate.


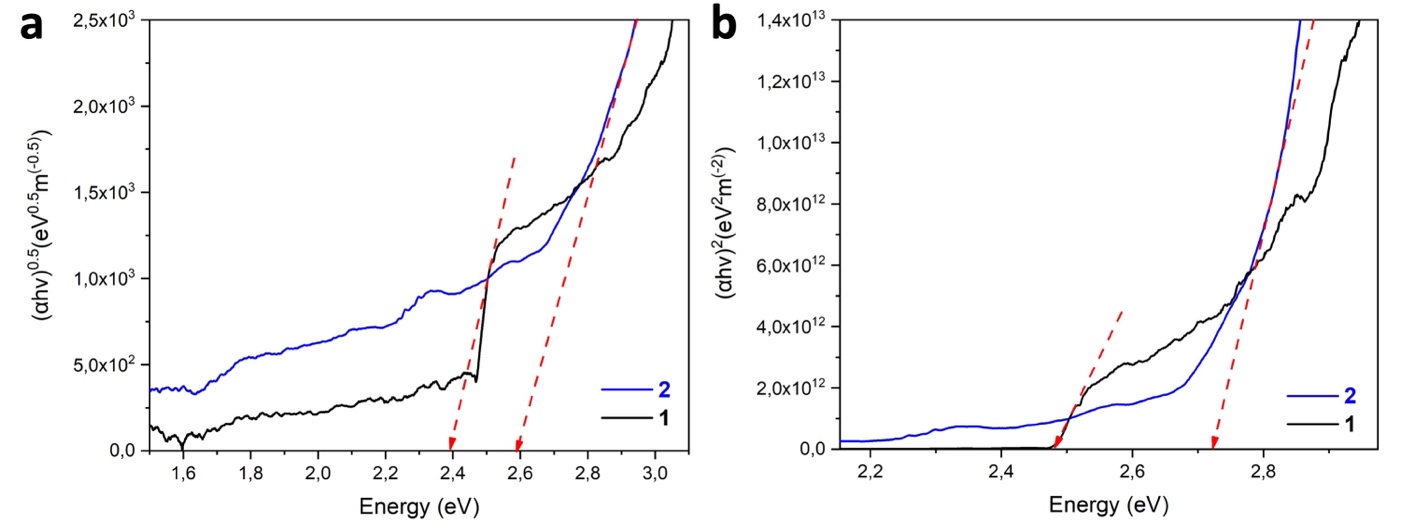


**Figure S12.** Tauc plots of **1** and **2**, reconstructed from Figure 4a: (a) indirect band gap; (b) direct band gap. The transformation from **1** to **2** is expressed as 9 ± 1 % blue shift of the bandgap of MOF.





**Figure S13.** Absorbance and reflectance Tauc plots of the diffuse reflectance ultraviolet-visible (DR UV-vis) spectroscopy of 2D Zn-NDC/BPE (**2**).


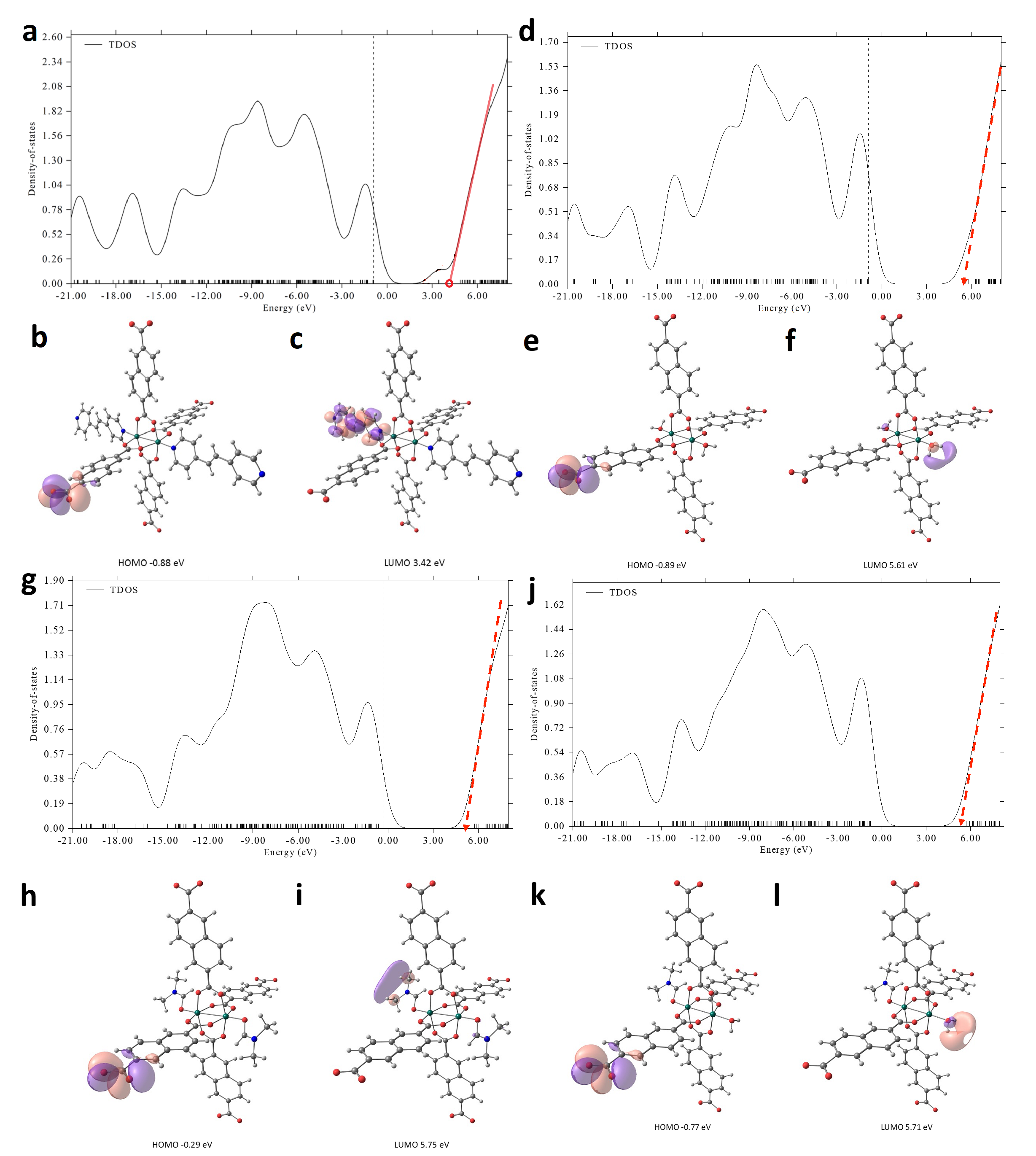


**Figure S14.** Total density-of-states (DOS) for **1** (a) and modeled **2** with two coordinated H_2_O molecules (d), two coordinated molecules of DMF (g), and with one molecule of H_2_O and DMF (j). Shape and energies of frontier molecular orbitals for **1** (b,c) and **2** MOF with different coordinated molecules (e,f,h,i,k,l). The transformation from **1** to **2** is expressed as 30 ± 10 % blue shift of the energy gap between HOMO/LUMO states of the MOF.

**Table S7.** Calculated values of ΔE (in kcal mol^-1^) for model transformations of Zn-NDC/BPE from 3D (**1**) to 2D (**2**) structure.

| Model structure | ΔE |
| --- | --- |
| 3D to 2D transformation by thermal treatment | |
| 3D MOF → 2D MOF | 68.08 |
| 3D to 2D transformation by solvent treatment | |
| 3D MOF + DMF + DMF → 2D MOF•2DMF | 60.84 |
| 3D MOF + H_2_O + H_2_O → 2D MOF•2H_2_O | 29.85 |

The estimated molecular formula of 2D MOFs with molecules of water: Zn_7_(NDC)_2_(BPE)_2-n_ ⋅nH_2_O and Co_7_(NDC)_2_(BPE)_2-n_ ⋅nH_2_O. The estimated molecular formula of 2D MOFs with molecules of DMF: Zn_7_(NDC)_2_(BPE)_2-n_ ⋅nDMF and Co_7_(NDC)_2_(BPE)_2-n_ ⋅nDMF.

**Table S8.** Raman peak analysis and assignments (Figures 2f, S16, S17). FWHM, full width at a half maxima.

| 3D Zn-NDC/BPE (**1**) | | 2D Zn-NDC/BPE (**2**) | | Assignment of Bonds | Ref. |
| --- | --- | --- | --- | --- | --- |
| Peak Position (cm^-1^) | FWHM (cm^-1^) | Peak Position (cm^-1^) | FWHM (cm^-1^) |  |  |
| 91 | 17,65 | 81 | 14,52 | Zn-O | [S36-S38] |
|  |  | 130 | 12,91 | Zn-O | [S36-S38] |
| 260 | 12,07 |  |  | Zn-O | [S39-S40] |
| 392 | 5,40 | 390 | 12,67 | Zn-N ν_st_ | [S41] |
| 524 | 6,25 | 512 | 6,29 | Out-of-plane def. vibr. | [S42] |
| 663 | 7,96 | 653 | 5,58 | In-plane bending of the ring | [S43] |
| 784 | 9,36 | 770 | 9,78 | ν_s_NC_3_ | [S44] |
| 861 | 8,42 | 844 | 7,69 | Naphthalene rings out-of plane vibr. | [S42] |
| 984 | 18,15 | 956 | 9,83 | s-m, C=C, C=N in plane vib. or arom. C-H in plane. def. vibr. | [S42] |
| 1032 | 7,74 | 1018 | 7,02 | Ring breathing | [S45] |
| 1148 | 9,37 | 1106 | 7,96 | arom. C-H in plane def. vibr. | [S42] |
|  |  | 1135 | 9,83 | arom. C-H in plane def. vibr. | [S42] |
| 1203 | 8,11 | 1189 | 7,84 | C_ring_-C_ethylene_ stretch coupled to ring distortion via bond bending asymmetrically | [S45] |
| 1243 | 9,08 | 1230 | 10,53 | Kekule vibrations  coupled to C_ring_-C_ethylene_  bend | [S45] |
| 1334 | 10,15 | 1302 | 8,08 | C_ring_-C_ethylene_ stretch coupled to ethylene stretching | [S45] |
|  |  | 1330 | 6,86 |  |  |
| 1396 | 16,43 | 1383 | 14,45 | Ring distortion via bond stretching, rings coupled asymmetrically or aromt. ring vibration | [S42, S45] |
| 1499 | 13,53 | 1472 | 12,81 | Ring distortion via bond bending asymmetrically/C=C, C=N in plane vib. or C-H sym. def. vibr. | [S7, S10] |
| 1538 | 9,16 | 1537 | 12,28 | Ring distortion via bond stretching/ | [S10] |
| 1615 | 8,72 | 1607 | 9,55 | Quinoidal modes coupled symmetrically | [S10] |
| 1637 | 7,88 | 1624 | 11,24 | Ethylene stretch/C=C str. vibration | [S7, S10] |

The Raman peak at 91 cm^-1^ in 3D structure of Zn-NDC/BPE (**1**) could be assigned with a basic phonon mode E2L. For 2D Zn-NDC/BPE (**2**), this mode shifted to 81 cm^-1^ with an appearance of a new mode at 130 cm^-1^, which could be assigned with a second order phonon mode of 2E2L.^S52^ Thus, the pronounced peak at ~130 cm^-1^ of the second order phonon mode 2E2L could indicate the structural transformation from 3D to 2D in our case (for instance, due to coordination of H_2_O molecules with the SBU, as we predicted by new calculations in Figure S14).

Raman peak at around 260 cm^-1^ is associated with Zn-O bonds, but intensity of this peak indicates the concentration of the nitrogen in SBU of Zinc.^S39, S40^ In our case, significant decreasing of this peak for **2**, compared with **1**, could be assigned with impairing the coordination of Zn-N bonds between Zn cluster and BPE ligand, which led to decreasing the concentration of nitrogen atoms in coordination sphere of 2D Zn-NDC/BPE (**2**).

Considering the profile of the Raman spectra in more detail, it was found that not only shifting to the lower energies was found for **2**, but also a full width at half maximum (FWHM, Table S8) values changed. Thus, values of FWHM for peaks, associated with Zn-O, decreasing, when transformation from the 3D structure (**1**) to the 2D (**2**), what indicates about the structural anharmonicity of 3D MOF in general and the coordination system in particular. At the same time, the peaks, which are associated with Zn-N bonds, at around 390 cm^-1^ for both **1** and **2** MOF has different FWHM values – 5,40 and 12,67 cm^-1^, which refers to the defects in the system of **2**. Weak Raman intensities of these peaks also decreasing from **1** to **2** during the transformation. Regarding the peaks associated with NDC ligand in the range of 520–1032 cm^-1^, FWHM values don’t differ much, but Raman intensities of peaks in the range 520–790 cm^-1^ increase. FWHM values of peaks of BPE ligands in the range of 1032 – 1637 cm^-1^, noticeably differ as well as Raman intensities. This also indicates an increase in the anharmonicity of the BPE ligand in **2** and an increase in its defectiveness after transformation from **1**.


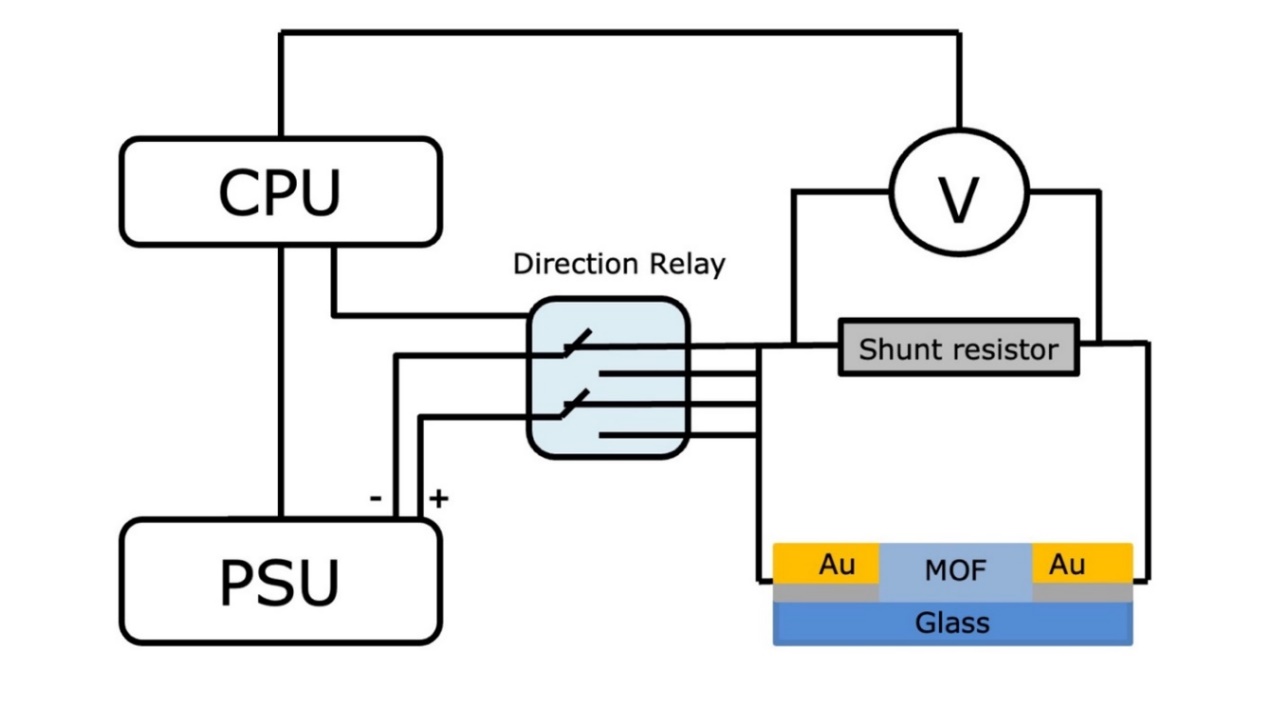


**Figure S15.** Scheme of resistive switching measurements: PSU – power supply unit; CPU – central processor unit.

AFM conductivity analysis:

$$R_{HRS}= \frac{U}{I}=\frac{1 V}{46*{10}^{-12}A}=2.1*{10}^{10} Ohm,$$

$$R_{LRS}= \frac{U}{I}=\frac{1 V}{989*{10}^{-12}A}={10}^{9} Ohm,$$

$$\frac{S}{d}=\frac{3.14*{(20*{10}^{-9})}^{2}}{45*{10}^{-9}}={27.9*10}^{-9} m,$$

$$\sigma_{HRS}= \frac{1}{58.59*10}=0.0017 S/m,$$

$$\sigma_{LRS}= \frac{1}{27.9}=0.036 S/m,$$

where voltage *U*, current *I*, resistance at high-resistive state *R_HRS_* and low-resistive state *R_LRS_*, surface area of conductive AFM tip *S*, thickness of the nanosheet *d* (45 nm), and conductivity in high-resistance *σ_HRS_* state and low-resistive state *σ_LRS_*. The estimated conductivity value (app. 10^-4^ S cm^-1^) ​​of the obtained nanosheets of **2** in the low-resistive state, on the one hand, is higher than that of most MOFs (in 3D and 2D state), and comparable with intentionally modified MOFs.^S46^ But, on the other hand, the estimated value of 10^-4^ S cm^-1^ is still inferior to the values ​​of ultrathin 2D MOFs and COFs with extremely small band gap.^S47,S48^


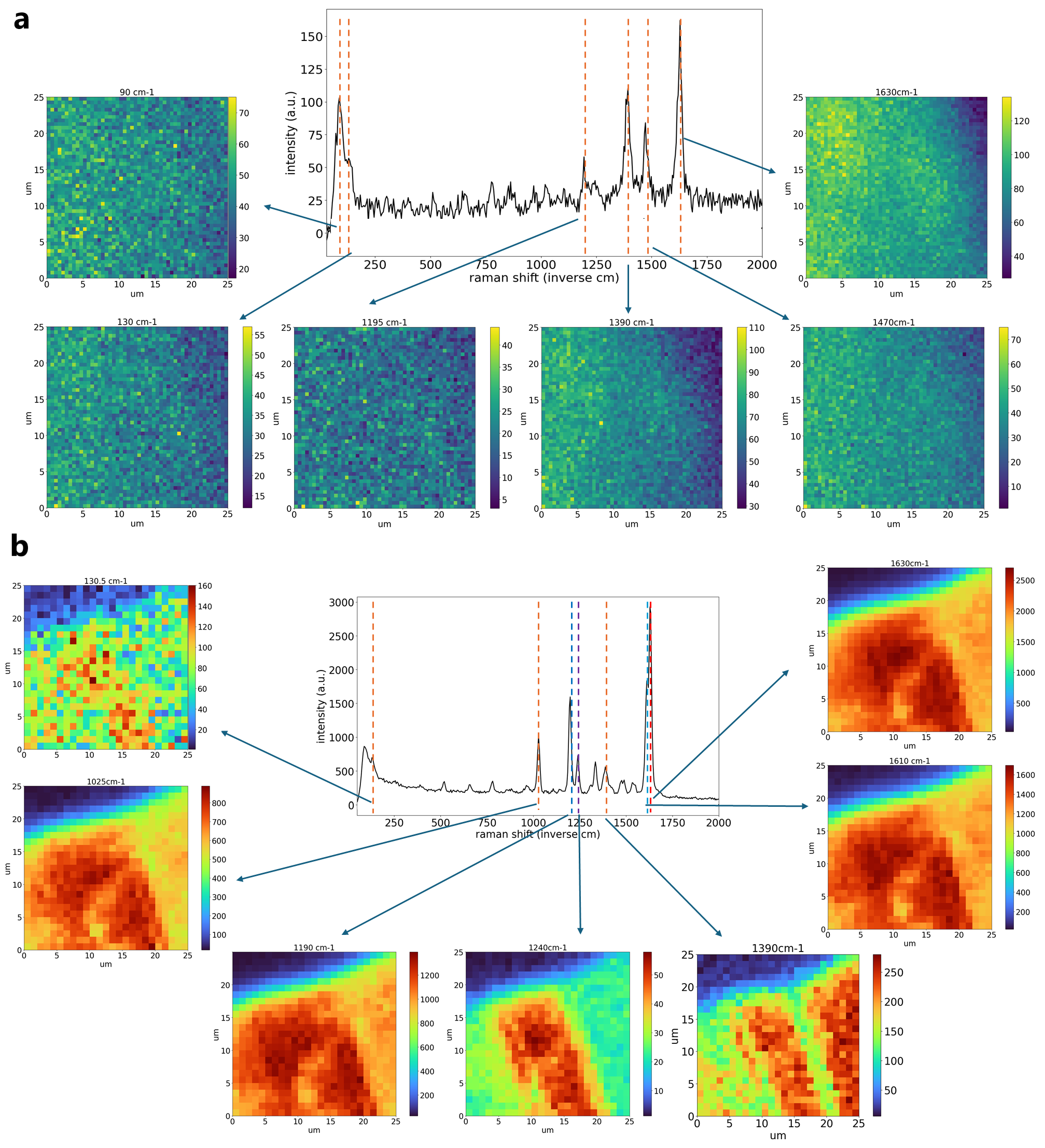


**Figure S16.** Confocal Raman mapping of single crystal of **1** (a) and **2** (b) over 25x25 µm area.


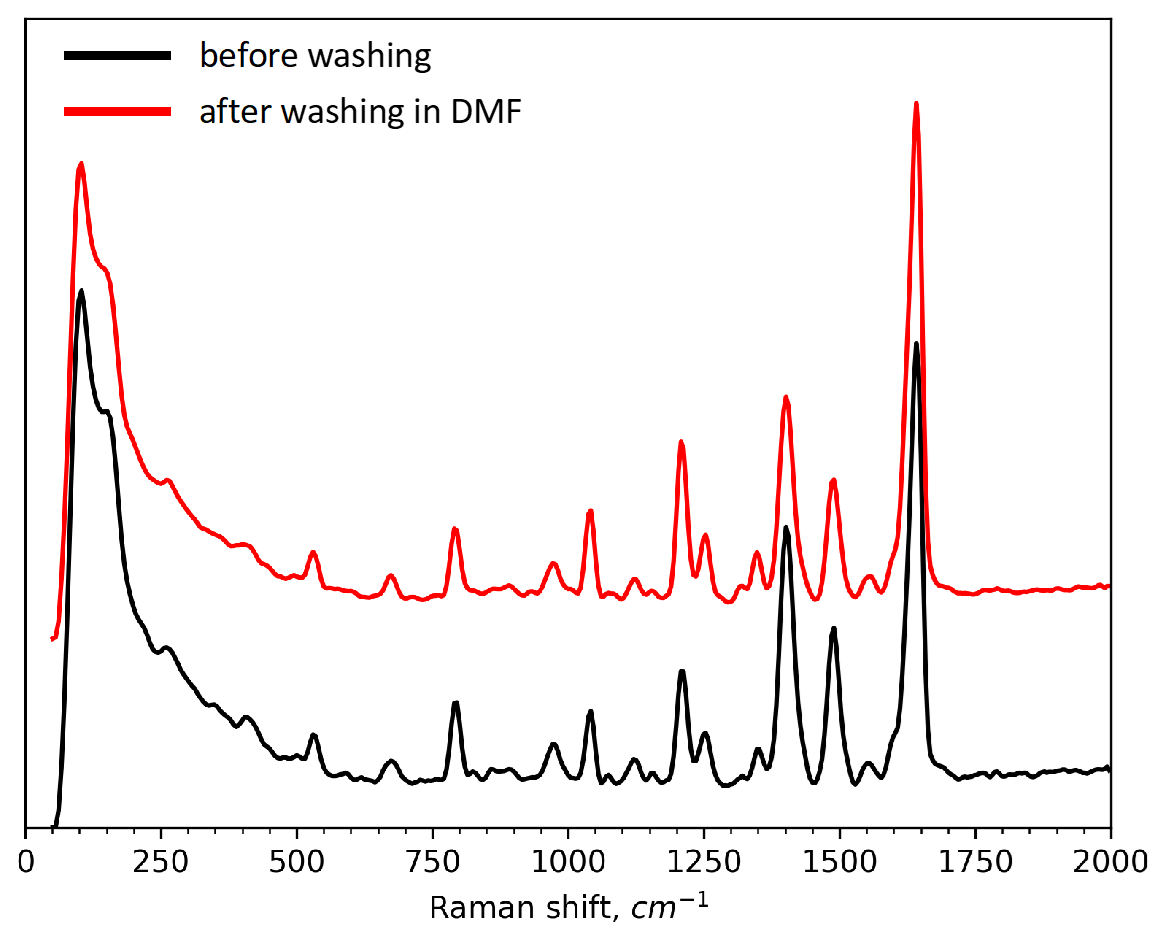


**Figure S17.** Normalized Raman spectra of 2D Zn-NDC/BPE (**2**) before and after additional washing in DMF.


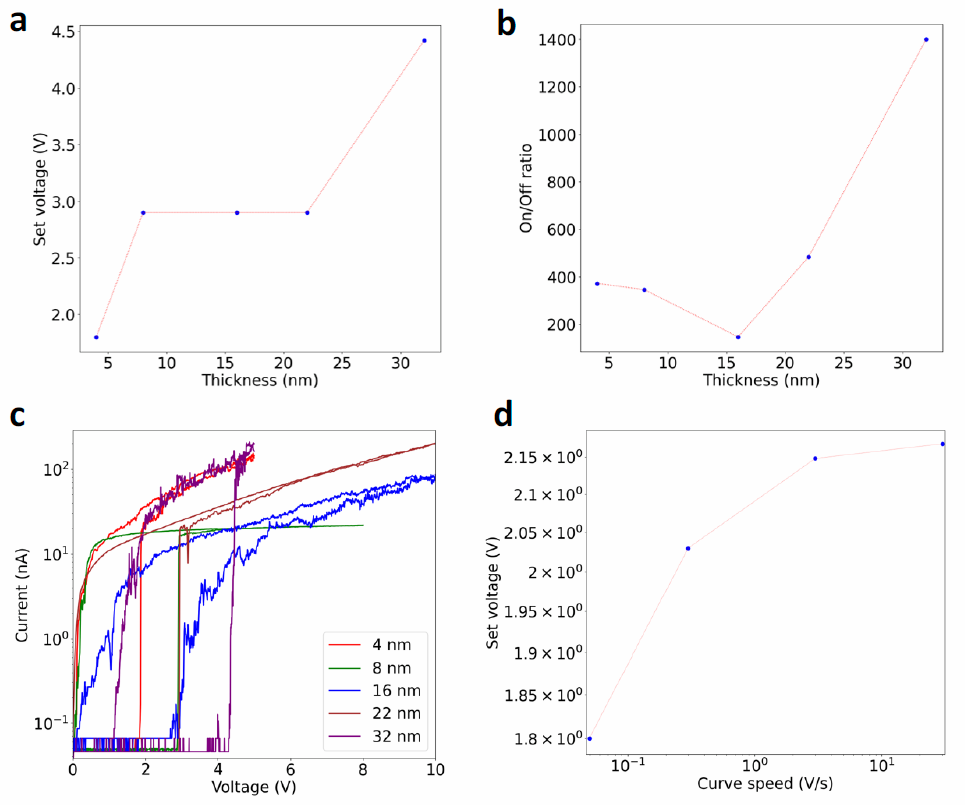


**Figure S18.** (a) Dependence of set voltage (V_set_) on the thickness of the nanosheets of **2**. (b) Dependence of ON/OFF ratio on the thickness of the nanosheets of **2**, read by 1.5 V. The nonlinear dependence can be explained by the residual BPE ligand, acting as a defect randomly from the nanosheet to another nanosheet. (c) Current-voltage curves for 4 to 32 nm nanosheets of **2** at a fixed ramp rate (1 V s^-1^) demonstrating the dependence of V_set_ and ON/OFF ratio on the nanosheet thickness. (d) Dependence of set voltage (V_set_) on the curve speed (ramp rate).

**Figure S19.** TEM analysis of 2D Zn-NDC/BPE (**2**) at 14.6 e^–^/Å^2^ per second. (a) and (b) TEM images of a fragment of **2** showing the degradation of the material upon low-dose of electron beam after 1 s of exposure.


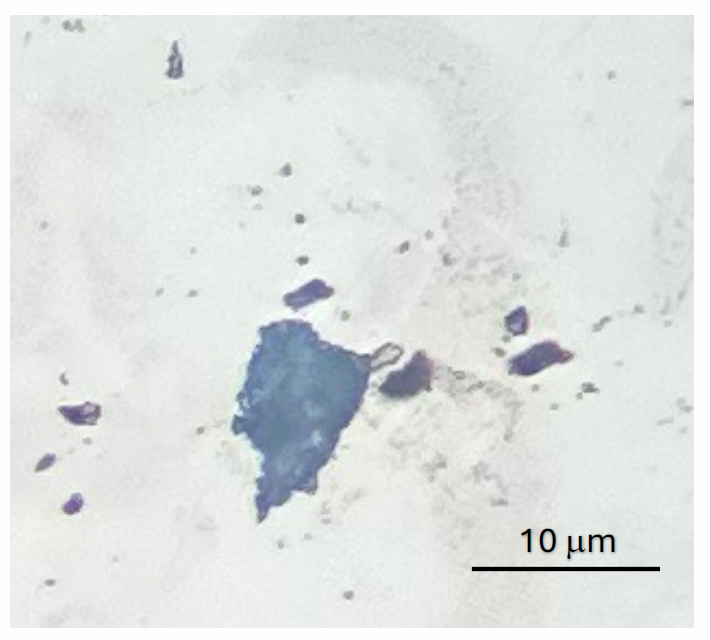


**Figure S20.** Optical image of **1’** after mechanical exfoliation process.

**References**

1. A. Kondo, T. Suzuki, R. Kotania, K. Maeda, Dalton Trans. 2017, 46, 6762-6768.
2. F. J. Claire, M. A. Solomos, J. Kim, G. Wang, M. A. Siegler, M. F. Crommie, T. J. Kempa, Nat. Commun. 2020, 11, 5524.
3. L. Wen, P. Cheng, W. Lin, **Chem. Commun.**, 2012, **48**, 2846-2848.
4. B. Ding, Y. Y. Wang, S. X. Liu, X. X. Wu, Z. Z. Zhu, J. Z. Huo, Y. Y. Liu, **CrystEngComm** 2015, **17**, 5396-5409.
5. J. Y. Choi, J. Flood, M, Stodolka, H. T. B. Pham, J. Park, ACS Nano 2022, 16, 3145–3151.
6. L. H. Wee, M. Meledina, S. Turner, G. Van Tendeloo, K. Zhang, L. M. Rodriguez-Albelo, A Masala, S. Bordiga, J. Jiang, J. A. R. Navarro, C. E. A. Kirschhock, J. A. Martens, J. Am. Chem. Soc. 2017, 139, 819–828.
7. S. Gao, R. Q. Fan, X. M. Wang, L. G. Wei, Y. Song, X. Du, K. Xing, P. Wanga, Y. L. Yang, **Phys. Chem. Chem. Phys.** 2016, **18**, 19001-19010.
8. Y. He, J. Shang, Q. Gu, G. Li, J. Li, R. Singh, P. Xiao, P. A. Webley, Chem. Commun. 2015, 51, 14716-14719.
9. T. Jadhav, Y. Fang, C.-H. Liu, A. Dadvand, E. Hamzehpoor, W. Patterson, A. Jonderian, R. S. Stein, D. F. Perepichka, J. Am. Chem. Soc. 2020, 142, 8862–8870.
10. A. Hazra, T. K. Maji, Inorg. Chem. 2020, 59, 12793–12801.
11. S. Khan, A. Frontera, R. Matsuda, S. Kitagawa, M. H. Mir, Inorg. Chem. 2022, 61, 3029–3032.
12. S. Oh, J. Park, M. Oha, IUCrJ 2019, 6, 681-687.
13. J. Hungwe, P. Tshuma, M. Gumbo, F. M. Amombo Noa, L. Öhrström, G. Mehlana, **CrystEngComm**, 2023, **25**, 1486-1494.
14. W. Pang, B. Shao, X.-Q. Tan, C. Tang, Z. Zhang, Jin Huang, Nanoscale 2020, 12, 3623-3629.
15. W. Pang, B. Shao, X. Chen, Q.-X. Gu, F.-J. Yang, S. Li, J. Huang, J. Colloid Interface Sci., 2022, 608, 306-312.
16. B. Shao, X. Chen, Y.-T. Xu, G.-Q. Li, J.-P. Zhong, T. Meng, Z. Zhang, F. P. Huang, J. Huang, J. Catal., **2022**, 413, 168-175.
17. M. Eddaoudi, J. Kim, D. Vodak, A. Sudik, J. Wachter, M. O’Keeffe, O. M. Yaghi, *Proc. Natl. Acad. Sci. U. S. A.* **2002**, 99, 4900–4904.
18. F. Shahangi Shirazi, K. Akhbari, *Inorganica Chim. Acta* **2015**, 436, 1–6.
19. M.C. Das, H. Xu, Z. Wang, G. Srinivas, W. Zhou, Y.-F. Yue, V.N. Nesterov, G. Qian, B. Chen, *Chem. Commun.* **2011**, 47 (42), 11715–11717.
20. S.-Y. Yang, H.-B. Yuan, X.-B. Xu, R.-B. Huang, *Inorganica Chim. Acta* **2013**, 403, 53–62.
21. M. Eddaoudi, J. Kim, N. Rosi, D. Vodak, J. Wachter, M. O’Keeffe, O.M. Yaghi, *Science* **2002**, 295 (5554), 469–472.
22. R.N. Devi, M. Edgar, J. Gonzalez, A.M.Z. Slawin, D.P. Tunstall, P. Grewal, P.A. Cox, P.A. Wright*, J. Phys. Chem. B* **2004**, 108 (2), 535–543.
23. J.J. Perry IV, P.L. Feng, S.T. Meek, K. Leong, F.P. Doty, M.D. Allendorf, *J. Mater. Chem.* **2012**, 22 (20), 10235–10248.
24. S. Zhou, W. Xu, P. Zhang, K. Tang, *Appl. Organomet. Chem.* **2021**, 35 (8), e6267.
25. N.L. Rosi, M. Eddaoudi, J. Kim, M. O’Keeffe, O.M. Yaghi, *Angew. Chemie Int. Ed.* **2002**, 41 (2), 284–287.
26. K.O. Kongshaug, H. Fjellvåg, *J. Solid State Chem.* **2004**, 177 (6), 1852–1857.
27. L. Ya-Min, W. Ying-Ying, L. Hai-Yan, Z. Xiao-Wei, J. Lin-Yu, D. Dong-Bin, *Chinese J. Struct. Chem.* **2019**, 38 (5), 761–768.
28. M.D. Won, Y.S. Su, L.C. U, L.C. Yeon, S.M. Gu, H.Y. Jeong, H.W. Seok, L.S. W., *Bull. Korean Chem. Soc.* **2001**, 22 (5), 531–533.
29. J. Kim, B. Chen, T.M. Reineke, H. Li, M. Eddaoudi, D.B. Moler, M. O’Keeffe, O. M. Yaghi, *J. Am. Chem. Soc.* **2001**, 123 (34), 8239–8247.
30. M. Dincǎ, J.R. Long, *J. Am. Chem. Soc.* **2005**, 127 (26), 9376–9377.
31. N. Saffon-Merceron, M.-C. Barthélémy, C. Laurent, I. Fabing, P. Hoffmann, A. Vigroux, *Zeitschrift für Anorg. und Allg. Chemie* **2016**, 642 (11–12), 709–713.
32. J.-H. Cho, S.M. Lee, J.W. Shin, D. Moon, K.S. Min, H.-I. Lee, *Korean Chem. Soc.* **2014**, 35 (3), 949–952.
33. N. Saffon-Merceron, A. Vigroux, P. Hoffmann, *Acta Crystallogr. Sect. E* **2019**, 75 (11), 1759–1762.
34. C. Hong, L. Li, J.-Y. Zou, L. Zhang, S.-Y. You, *Dalton Trans.* **2023**, 52 (18), 6067–6076.
35. B. Chen, S. Ma, F. Zapata, E.B. Lobkovsky, J. Yang, *Inorg. Chem.* **2006**, 45 (15), 5718–5720.
36. A. Jagannatha Reddy, M.K. Kokila, H. Nagabhushana, J.L. Rao, C. Shivakumara, B.M. Nagabhushana, R.P.S. Chakradhar, *Spectrochim. Acta A* **2011**, 81, 53.
37. B. Hadžić, N. Romčević, D. Sibera, U. Narkiewicz, I. Kuryliszyn-Kudelska, W. Dobrowolski, M. Romčević, *J. Phys. Chem. Solids* **2016**, 91, 80.
38. M. Silambarasan, S. Saravanan, T. Soga, *Int.J. ChemTech Res.* **2015**, 7, 1644-1650.
39. W. W. Liu, B. Yao, Z. Z. Zhang, Y. F. Li, B. H. Li, C. X. Shan, J. Y. Zhang, D. Z. Shen, X. W. Fan, *J. Appl. Phys.* **2011,** 109, 093518.
40. A. Kaschner, U. Haboeck, M. Strassburg, M. Strassburg, G. Kaczmarczyk, A. Hoffmann, C. Thomsen, A. Zeuner, H. R. Alves, D. M. Hofmann, B. K. Meyer, *Appl. Phys. Lett*. **2002**, 80, 1909–1911.
41. G. R. Cayley, D. N. Hague, *Trans. Faraday Soc.* **1971**, 67, 2896.
42. A. Modrow, D. Zargarani, R. Herges, N. Stock, *Dalton Trans*. **2011**, 40, 4217.
43. X. Yang, Y. Zhang, F. Li, T. Guo, Y. Wu, F. Jin, M. Fang, Y. Lan, Y. Li, Y. Zhou, Z. Zoub, ***Dalton Trans.,*** **2017, 46,** 8204-8218.
44. L. Abylgazina, I. Senkovska, S. Ehrling, V. Bon, P. St. Petkov, J. D. Evans, S. Krylova, A. Krylov, S. Kaskel, *CrystEngComm*, **2021**, 23, 538-549.
45. E. A. Sprague-Klein, B. Negru, L. R. Madison, S. C. Coste, B. K. Rugg, A. M. Felts, M. O. McAnally, M. Banik, V. A. Apkarian, M. R. Wasielewski, M. A. Ratner, T. Seideman, G. C. Schatz, R. P. Van Duyne, *J. Am. Chem. Soc.* **2018**, 140, 10583−10592.
46. L. S. Xie, G. Skorupskii, M. Dincă, *Chem. Rev*. **2020**, 120, 8536–8580.
47. R. Dong, P. Han, H. Arora, M. Ballabio, M. Karakus, Z. Zhang, C. Shekhar, P. Adler, P. St. Petkov, A. Erbe, S. C. B. Mannsfeld, C. Felser, T. Heine, M. Bonn, X. Feng, E. Cánovas, *Nat. Mater.* **2018**, 17, 1027–1032.
48. D. Feng, T. Lei, M. R. Lukatskaya, J. Park, Z. Huang, M. Lee, L. Shaw, S. Chen, A. A. Yakovenko, A. Kulkarni, J. Xiao, K. Fredrickson, J. B. Tok, X. Zou, Y. Cui, Z. Bao, *Nat. Energy* **2018**, 3, 30–36.
49. Y.-P. Wei, Y.-W. Zhang, J.-S. Chen, C.-J. Mao, B.-K. Jin, *Mikrochim. Acta* **2020**, 187, 455.
50. Y. Liu, C. Wang, S. Ju, M. Li, A. Yuan, G. Zhu, *Prog. Nat. Sci.: Mater. Int.* **2020**, 30, 185-191.
51. G. S. Kim, Y. Lim, J. Shin, J. Yim, S. Hur, H.-C. Song, S.-H. Baek, S. K. Kim, J. Kim, C.-Y. Kang, J.-S. Jang, *Adv. Sci*. **2023**, 10, 2301002.
52. M. Silambarasan et al ,. *Int. J. Chem Tech Res*. **2014-2015**, 7, 1644-1650.
